# Myeloid cell genome-wide screen identifies variants associated with *Mycobacterium tuberculosis*–induced cytokine transcriptional responses

**DOI:** 10.1172/JCI179822

**Published:** 2025-05-22

**Authors:** Joshua J. Ivie, Kimberly A. Dill-McFarland, Jason D. Simmons, Glenna J. Peterson, Penelope H. Benchek, Harriet Mayanja-Kizza, Lily E. Veith, Moeko Agata, Dang T.M. Ha, Ho D.T. Nghia, W. Henry Boom, Catherine M. Stein, Chiea C. Khor, Guy E. Thwaites, Hoang T. Hai, Nguyen T.T. Thuong, Xuling Chang, Sarah J. Dunstan, Thomas R. Hawn

**Affiliations:** 1 Department of Global Health and; 2 Department of Medicine, University of Washington, Seattle, Washington, USA.; 3 Department of Population and Quantitative Health Sciences, Case Western Reserve University, Cleveland, Ohio, USA.; 4 Department of Medicine, School of Medicine, Makerere University, Kampala, Uganda.; 5 Pham Ngoc Thanh Hospital, Ho Chi Minh City, Vietnam.; 6 Oxford University Clinical Research Unit, Ho Chi Minh City, Vietnam.; 7 Hospital for Tropical Diseases, Ho Chi Minh City, Vietnam.; 8 Pham Ngoc Thach University of Medicine, Ho Chi Minh City, Vietnam.; 9 Department of Medicine, Case Western Reserve University, Cleveland, Ohio, USA.; 10 Genome Institute of Singapore, A-STAR, Singapore.; 11 Singapore Eye Research Institute, Singapore National Eye Centre and Eye ACP, Duke–National University of Singapore, Singapore.; 12 Department of Biochemistry, National University of Singapore, Singapore.; 13 Centre for Tropical Medicine and Global Health, Nuffield Department of Medicine, University of Oxford, Oxford, United Kingdom.; 14 Department of Infectious Diseases, University of Melbourne at the Peter Doherty Institute for Infection and Immunity, Parkville, Victoria, Australia.

**Keywords:** Genetics, Infectious disease, Cytokines, Genetic variation, Tuberculosis

## Abstract

Immune and clinical outcomes to *Mycobacterium tuberculosis* (Mtb) infection vary greatly between individuals, yet the underlying genetic and cellular mechanisms driving this heterogeneity remain poorly understood. We performed a cellular genome-wide association study to identify genetic variants associated with Mtb-induced monocyte transcriptional expression of *IL1B*, *IL6*, *TNF*, and *IFNB1* via RNA-Seq in a Ugandan cohort. Significantly associated variants were assessed for transferability in an independent Seattle cohort, further validated in vitro, and assessed for clinical phenotype associations. We identified 77 loci suggestively associated with Mtb-induced cytokine expression in monocytes in Uganda. SNPs associated with Mtb-induced *TNF* were enriched within α-linolenic acid metabolism pathway genes, which was validated in vitro using PLA2 inhibitors. Four loci maintained significant associations in Seattle. We validated a cytokine effect with siRNA knockdown for two of these loci, which mapped to the genes *SLIT3* and *SLC1A1*. Furthermore, exogenous treatment of macrophages with SLIT3 enhanced Mtb intracellular replication. Finally, *SLC1A1* and *SLIT3* variants were associated with susceptibility to tuberculous meningitis and subsequent survival, respectively, in a Vietnamese cohort. In summary, we identified multiple variants and pathways associated with Mtb-induced cytokine transcriptional responses that were validated in vitro and were associated with clinical tuberculosis susceptibility.

## Abstract 2

Genetic variants regulating the *Mycobacterium tuberculosis*-induced cytokine response pinpoint genes and pathways that modulate *in vitro* human myeloid cell responses and clinical Tuberculosis susceptibility.

## Introduction

Tuberculosis (TB) is a leading cause of death worldwide, including 1.3 million deaths in 2022 (1). Clinical outcomes after exposure to *Mycobacterium tuberculosis* (Mtb) are varied and include pathogen clearance, asymptomatic infection, pulmonary TB, and tuberculous meningitis (TBM) (2). Although parts of the heterogeneity of clinical outcomes are attributable to known clinical risk factors, the majority of mechanisms are poorly understood (3). One hypothesis is that genetic variation in the host immune response regulates the pleiotropic outcomes after Mtb exposure. Mtb has a long coevolutionary history with humans allowing for the accumulation of numerous genetic adaptations in both pathogen and host that regulate pivotal responses (4, 5). Defining the genetic variants responsible for controlling the Mtb-induced host response may yield important insights into differences in host progression and identify novel areas of Mtb-response biology for further research.

Several lines of evidence suggest that genetic factors regulate susceptibility to Mtb infection and TB disease (6–19). Mendelian studies in pediatric populations support an important role of IL-12/IFN-γ–mediated pathways for controlling mycobacterial infection (20). Several variants have been associated with pulmonary TB across a number of genome-wide association studies (GWAS) (8, 12–19). However, despite numerous findings of genome-wide significance, there is little concordance between studies. Although susceptibility to TB clinical outcomes has consistently been found to be highly heritable, the variants previously identified only explain a small portion of the genetically indicated effect (8, 9, 11). Potential reasons for this missing heritability include cohort sample size, heterogeneity of clinical phenotypes, lack of adjustment for non-genetic risk factors, and Mtb strain diversity. A cellular GWAS approach addresses these limitations by examining genetic variants associated with inter-individual intermediate traits measured by in vitro assays (21). Unencumbered by many of the same limitations of clinical studies, variants regulating essential pathogen-induced cellular traits can be identified. These variants can then be used to identify important host response factors and further elucidate previous clinical GWAS findings by yielding mechanistic insight. This technique has been used to characterize the genetic regulation of multiple cellular phenotypes, including cytokine production in response to a variety of microbial stimuli, cell death, and intracellular replication of infections such as *Salmonella* and *Chlamydia* (21–28).

Macrophages are pivotal innate immune cells in TB pathogenesis owing to their early roles in initial detection of Mtb, serving as a cellular home for persistent infection, and involvement in clearance of infection (2, 29). Macrophage signaling pathways are important at each of these steps to modulate pathogenesis. In particular, the host cytokine response plays a large role in determining infection outcomes, requiring a sufficient response to control infection without over-responding and causing tissue pathology (30). Interleukin-1β (*IL1B*), interleukin-6 (*IL6*), tumor necrosis factor (*TNF*), and interferon-β (*IFNB1*), as key components of this response, have important functions in initial detection of Mtb, controlling infection, initiating effective T cell responses, and modulating immunopathology (31–40). To our knowledge, genome-wide assessment of genetic regulation of live virulent Mtb-induced cytokine responses in macrophages has not been assessed previously.

In the current study, we characterized the cytokine transcriptional profile of monocytes and macrophages isolated from two distinct human population cohorts in response to live Mtb infection in vitro and performed genome-wide assessment of the major genetic variants and genetically regulated pathways associated with differential Mtb-induced cytokine response. The genes, *SLIT3* and *SLC1A1*, and the α-linolenic acid signaling pathway were identified as candidate response regulators and were validated using in vitro macrophage assays. Lastly, we investigated whether the *SLIT3* and *SLC1A1* variants affecting the Mtb-induced cytokine response were further associated with clinical TB phenotypes and found significant differences in TBM susceptibility and subsequent survival.

## Results

### Mtb-induced cytokine GWAS yields 77 genomic loci with suggestive associations.

**Figure 1:**
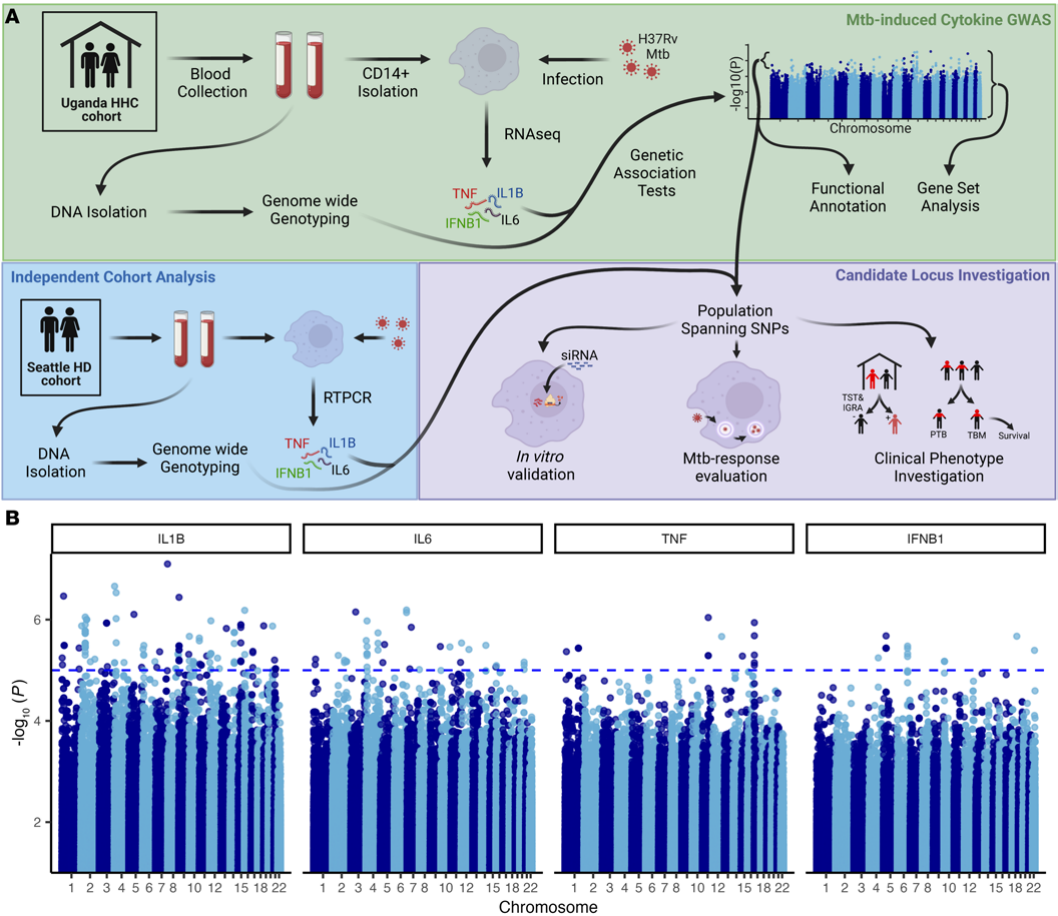
GWAS revealed 77 distinct genomic loci suggestively associated with Mtb-induced cytokine induction. (**A**) Diagram of workflow for the cellular GWAS approach and subsequent validation and mechanistic investigation. HHC indicates household contact cohort and HD indicates healthy donor cohort. Created with BioRender (biorender.com). (**B**) Manhattan plot shows GWAS results of all 4 cytokines plotted for –log_10_(*P* values). Results were calculated with a linear mixed model in GENESIS to account for population structure, genetic relatedness, sex, age, and RNA-Seq batch (Supplemental Figure 2). Dashed horizontal line indicates suggestive threshold, *P* < 1 × 10^–5^.

To discover genetic variants associated with differential Mtb-induced cytokine production, we examined 100 individuals in a Ugandan cohort with a previously generated RNA-Seq dataset of CD14^+^ monocytes infected for 6 hours with H37Rv Mtb compared with media control (Figure 1A and Supplemental Table 1; supplemental material available online with this article; https://doi.org/10.1172/JCI179822DS1) (41, 42). We examined *IL1B*, *IL6*, *TNF*, and *IFNB1* log_2_ fold change in expression levels in this dataset, which were all highly increased during Mtb infection (Supplemental Figure 1A). All 100 individuals were simultaneously genotyped and imputed, yielding 8.3 million testable SNPs. We assessed each SNP using a linear model in the GENESIS package in R, adjusting for sex, age, and batch, and controlled for ancestry and population structure by adjusting for genotypic principal components (PCs), PC1, PC2, and kinship (Supplemental Figure 2). We then clustered individual SNP results into independent genomic loci using linkage disequilibrium–based (LD-based) clumping to the most significant, lead SNP within the locus. We did not identify any signal at a genome-wide level of significance (*P* < 5 × 10^–8^). However, a total of 77 genomic loci reached suggestive significance level (*P* < 1 × 10^–5^), including 51 for Mtb-induced *IL1B*, 24 for *IL6*, 6 for *TNF*, and 6 for *IFNB1* (Figure 1B). Of the 77 total loci, 10 were found to have a suggestive association for both Mtb-induced *IL1B* and *IL6* simultaneously. The majority of SNPs were associated with decreased Mtb-induced cytokine expression (Supplemental Table 2). In post hoc analyses, lead SNPs of many of these loci were found to be additionally associated with baseline, media cytokine expression (Supplemental Table 2). Although assessed independently, Mtb-induced *IL1B* expression, *IL6* expression, and *TNF* expression were highly correlated (*R* = 0.51–0.8), and SNPs with a suggestive association with one cytokine often had some association in the other two (Supplemental Figure 1, B and C). In summary, 77 genomic loci were identified as candidate genetic regulators of the Mtb-induced cytokine response in a Ugandan cohort.

### Functional annotation of candidate loci reveals potential mechanism and history of phenotypic effect.

To identify potential mechanisms of genetic effect, we mapped each of the 77 loci to proximal genes, assessed expression quantitative trait loci (eQTL) effects, predicted deleterious SNPs, and identified previous locus associations in the literature and GWAS catalog (Table 1 and Supplemental Table 2) (43, 44). Many of the mapped genes have previously been associated with cytokine regulation and Mtb response (45–53). We further assessed potential *cis*-eQTL effects of loci SNPs on baseline, media expression of adjacent genes within 250 kb and identified several SNPs significantly associated with *cis*-eQTL effects (Supplemental Figure 3A). Additionally, many loci have SNPs with Combined Annotation Dependent Depletion (CADD) scores ranking them from the top 10% to 1% most deleterious SNPs (CADD = 10–20) (Supplemental Figure 3B). Lastly, multiple SNPs had previous COVID-19, autoimmune disease, protein QTL, and myeloid cell phenotype associations within the GWAS catalog. Together, these analyses demonstrate that many of the genomic loci identified have additional forms of evidence that support their potential to have an effect on Mtb-induced cytokine expression.

### Gene set analysis: MAGMA identifies pathways enriched for Mtb-induced TNF effect.

**Figure 2:**
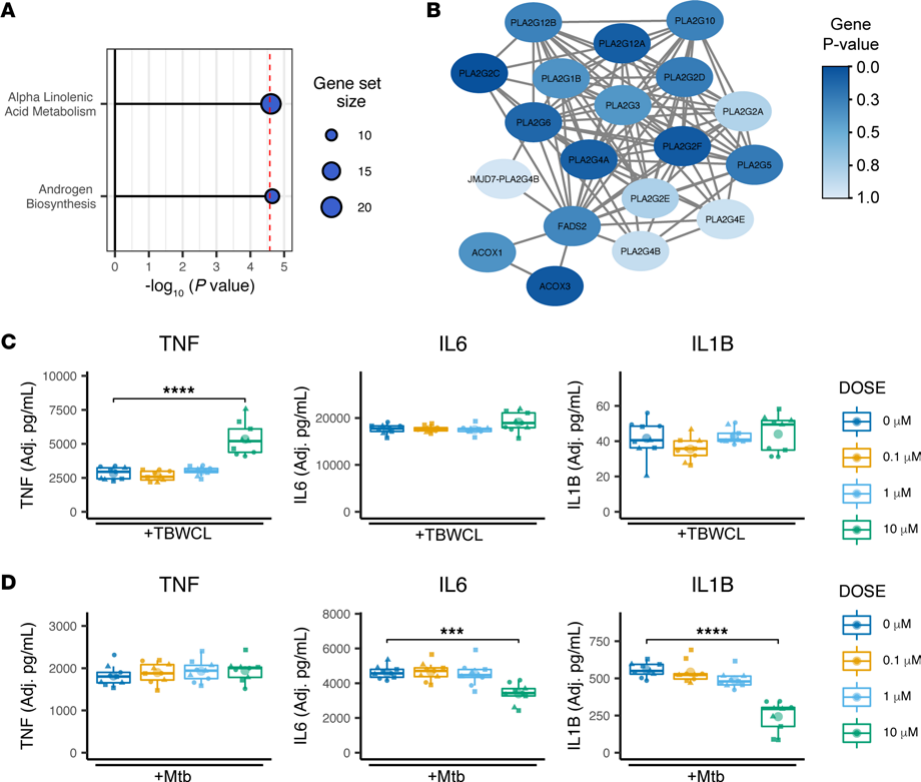
MAGMA enrichment identifies Mtb-induced *TNF* association with α-linolenic acid metabolism. (**A**) Previously calculated SNP *P* values for each cytokine were used to calculate gene-level *P* values, mapped to within each gene, accounting for SNP LD using MAGMA multi-model. Selected C2-canonical pathways (KEGG, Reactome, BioCarta, and Pathway Interaction Database) and C5-GO terms were assessed. Significant results after Bonferroni multiple correction are shown. Dashed vertical line indicates threshold for significance (adjusted *P* value < 0.05). (**B**) Network of genes within α-linolenic acid metabolism gene set was annotated for interaction using STRINGdb and plotted for MAGMA-calculated gene *P* value significance showing multiple PLA2 isoforms with increased significance. (**C** and **D**) TBWCL (**C**) and Mtb-induced secretion (**D**) of TNF, IL1B, and IL6 assessed by ELISA after increasing doses of cPLA2 inhibitor AACOCF3 pretreatment in human macrophages show that PLA2 enzymes do significantly affect TBWCL-induced TNF response and Mtb-induced IL1B and IL6 response. Results shown are from 3 biological replicates in 3 human donors per treatment. Significance was assessed using linear mixed model adjusting for random effect of donor followed by ANOVA and pairwise comparisons. Individual data points were plotted according to model calculated values adjusted for donor intercept. ***P < 0.001, ****P < 0.0001. Individual donor values are presented in Supplemental Figure 6A.

We performed gene set analysis (GSA) using Multi-marker Analysis of GenoMic Annotation (MAGMA), to determine whether SNPs had a significant joint association with Mtb-induced cytokine response in particular gene sets within the Molecular Signatures Database (MSigDB) (54–56). Gene *P* values were first determined from the aggregate signal of SNPs located within gene boundaries and adjusted for multiple correction while accounting for linkage disequilibrium (LD). GSA was then performed for each cytokine independently. Overall, across all gene sets and all 4 cytokines, only two significant associations were found, both of which were canonical pathways enriched for genes associated with Mtb-induced *TNF* (Figure 2A). One of these two significant pathways was α-linolenic acid metabolism, which has been implicated in direct effects on mycobacterial growth in addition to other macrophage response phenotypes (57–59). Analysis of the 19 gene-level *P* values and genotypic mean plots within the gene set identified early QQ plot deviation driven by numerous independent SNPs, indicating that significance marks a true gene set and multiple-SNP effect rather than being due to outlier effects (Supplemental Figures 4 and 5). Network visualization demonstrated that much of the α-linolenic acid metabolism pathway gene set is composed of a variety of PLA2 family members, which are known to regulate inflammation (60). The more significant gene-level *P* values appeared to be spread among multiple unrelated PLA2 isozymes within the pathway (Figure 2B). We next examined whether in vitro perturbation of PLA2 activity modulated the Mtb-induced cytokine response. Because of the previously identified correlation of *IL1B*, *IL6*, and *TNF* levels, we assessed the composite effect on these 3 inflammatory cytokines simultaneously. We used both live Mtb and Mtb whole-cell lysate (TBWCL) as stimuli to examine different activation pathways. We first compared the effects of the cytosolic PLA2 inhibitor AACOCF3 on macrophage cytokine production in response to TBWCL and identified a highly significant effect on Mtb-induced TNF secretion at the 10 μM dose (Figure 2C; *P* < 0.0001). No significant effect on IL1B or IL6 was observed. In contrast, upon live Mtb infection, we found that macrophage production of TNF was not significantly affected, whereas IL1B and IL6 were significantly decreased (Figure 2D; *P* < 0.001). In summary, these data showed that the aggregate effect of SNPs associated with Mtb-induced *TNF* expression was found to be significantly enriched in genes of the α-linolenic acid metabolism pathway. Further investigation of this gene set identified PLA2 as a major genetic regulator of Mtb-induced cytokine production, which was confirmed with cellular studies.

### Multiple candidate loci have a population-spanning effect in an independent cohort.

**Figure 3:**
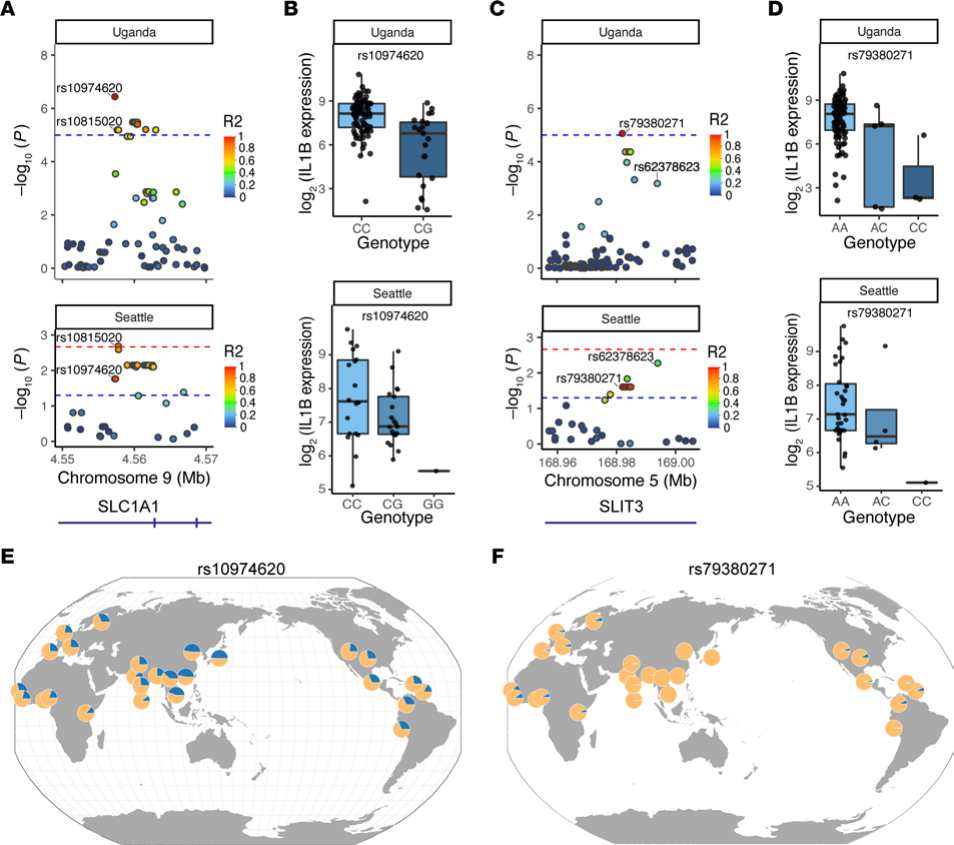
Multiple SNPs associated with Mtb-induced cytokine expression in the Uganda cohort show association with cytokine expression in a Seattle population cohort. Lead SNPs that passed the suggestive threshold for a particular cytokine within the Uganda population (*n* = 100) were tested for association with the corresponding cytokine in the Seattle population (*n* = 40). Four loci were associated with cytokine expression in the 2 populations (*P* < 0.05). To account for differences between populations, all SNPs within the 2 loci were assessed for linkage disequilibrium (LD) and association with corresponding cytokine expression. (**A**) *P* values of SNPs of the chr 9p24.2 locus, within the gene *SLC1A1*, with coloration of individual SNP points indicating LD with rs10974620 within each population. (**B**) Differences in Mtb-induced *IL1B* expression are plotted according to genotype for the chr 9p24.2 locus lead SNP of the Uganda population, rs10974620. (**C**) *P* values of SNPs of the chr 5q34 locus, within the gene *SLIT3*, with coloration of individual SNP points indicating LD with rs79380271 within each population. (**D**) Differences in Mtb-induced *IL1B* expression are plotted according to genotype for the chr 5q34 locus lead SNP of the Uganda population, rs79380271. Threshold lines indicate nominal *P* value threshold (blue, *P* < 0.05 unadjusted) and multiple corrected threshold (red, 0.05/number of SNPs tested for each cytokine). For both loci, multiple SNPs are significantly associated with decreased Mtb-induced *IL1B* expression in both populations. (**E** and **F**) Global minor allele frequency of the *SLC1A1* locus Uganda sentinel SNP, rs10974620 (**E**), and *SLIT3* locus Uganda sentinel SNP, rs79380271 (**F**), plotted using Geography of Genetic Variants browser.

Since genetic effects can be population specific, we next examined whether any of the Uganda suggestive loci were associated with Mtb-induced cytokine production in an independent cohort. Using a cohort of 40 Seattle healthy donors, we stimulated monocyte-derived macrophages with Mtb or media alone and measured cytokine expression after 6 hours via quantitative PCR. The lead SNP of each locus suggestively associated with Mtb-induced cytokine expression in the Uganda cohort was examined in the Seattle cohort for association with Mtb-induced cytokine expression. Of the 77 SNPs in the original Uganda population, over half were unavailable to assess because of differences in SNP imputation and allele frequency between the Uganda and Seattle cohorts. However, of the remaining SNPs, 3 SNPs (rs10974620, rs79380271, and rs11881732) were found to be significantly associated with Mtb-induced *IL1B* expression and 1 SNP (rs16947739) significantly associated with Mtb-induced *TNF* expression at a nominal, *P* < 0.05 threshold (Supplemental Table 3). Because of differences in the number of loci tested per cytokine, only the SNP for Mtb-induced *TNF* (rs16947739) surpassed the significance threshold for multiple correction of number of SNPs tested. To account for differences between populations, all SNPs mapping within the 4 associated loci were assessed for LD and association with cytokine expression. Two of the 4 loci, located at chromosome (chr) 9p24.2 and chr 5q34, showed a number of SNPs in LD that were significantly associated with reduced Mtb-induced *IL1B* in both populations (Figure 3, A–D, and Supplemental Figure 7). The lead SNPs of the 2 loci (chr 9p24.2 and chr 5q34) were different in the 2 populations; rs10974620 and rs10815020 are the sentinel SNPs for the chr 9p24.2 loci, and rs79380271 and rs62378623 are the sentinel SNPs for the chr 5q34 loci, for Uganda and Seattle, respectively. The sentinel SNPs for Uganda are present in the majority of populations globally, with the exception of some parts of Asia for rs79380271 (Figure 3, E and F). Functional annotation revealed that the sentinel SNPs for the chr 9p24.2 and chr 5q34 loci were located within introns of the genes *SLC1A1* and *SLIT3*, which have previously been linked to cytokine regulation in the literature (47, 48). Together, these data suggest that *SLIT3* and *SLC1A1* are lead candidate genes as major regulators of Mtb-induced *IL1B* in humans.

### SLC1A1 and SLIT3 affect Mtb-induced cytokine induction.

**Figure 4:**
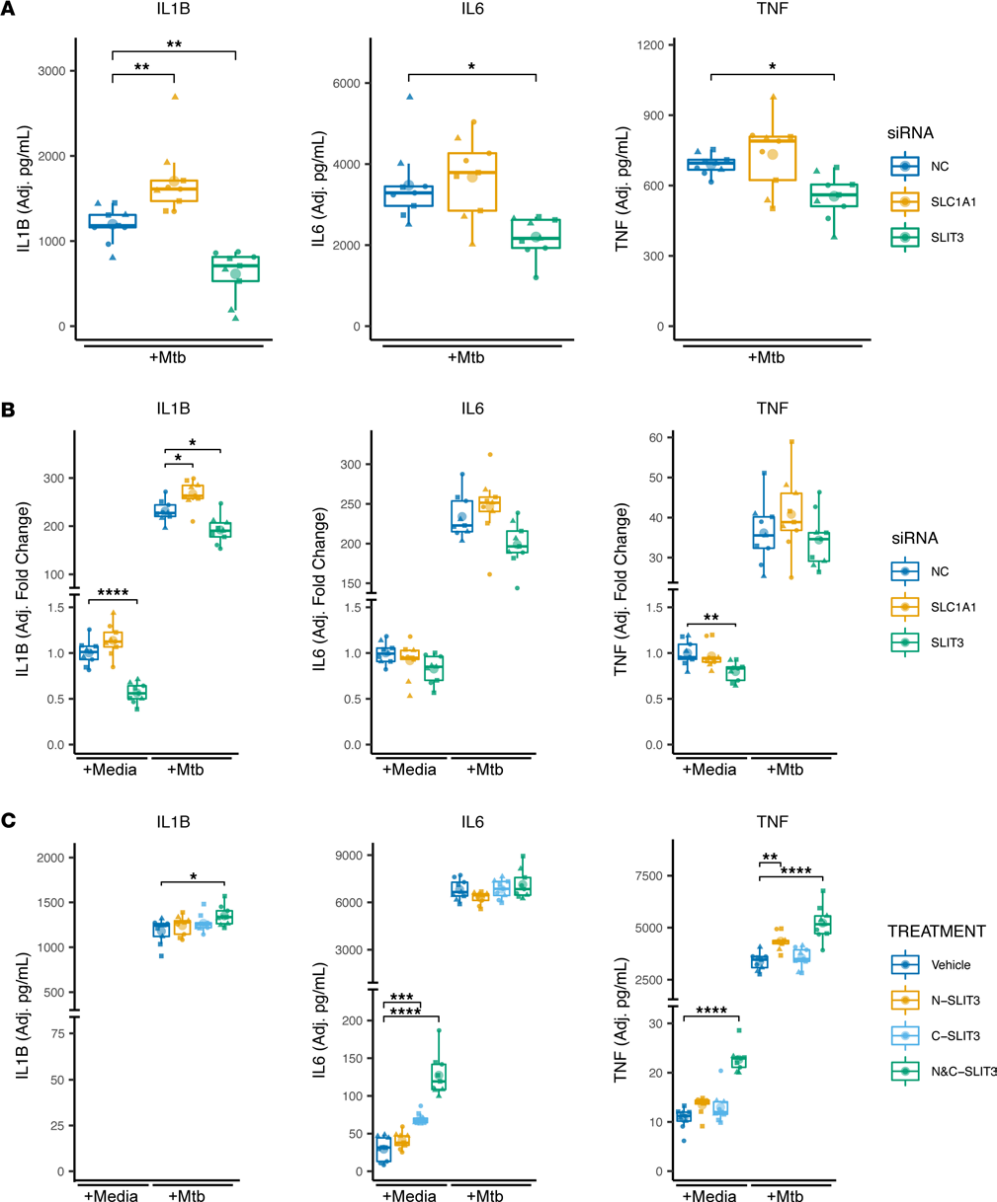
In vitro investigation of SNP-associated genes identifies *SLC1A1* and *SLIT3* as major regulators of the Mtb-induced cytokine response. (**A** and **B**) siRNA knockdown of *SLC1A1* and *SLIT3* expression was used to determine effect on IL1B, IL6, and TNF secretion measured by ELISA after 24 hours of Mtb infection (MOI = 1) (**A**) and *IL1B*, *IL6*, and *TNF* mRNA expression after 6-hour mock and Mtb infection (**B**). (**C**) Purified hSLIT3 N- and C-terminal fragments were tested for effect on IL1B, IL6, and TNF secretion measured by ELISA after 24-hour mock and Mtb infection. Results are from 3 biological replicates in 3 human donors per siRNA or treatment. ND, not detected. Significance was assessed using linear mixed model adjusting for random effect of donor followed by ANOVA and pairwise comparisons. Individual data points were plotted according to model calculated values adjusted for donor intercept. *P < 0.05, **P < 0.01, ***P < 0.001, ****P < 0.0001. Individual donor values are presented in Supplemental Figure 6, B–D.

To experimentally validate whether the identified candidate genes had an effect in vitro, we used siRNA in monocyte-derived macrophages (MDMs) and examined the effects of *SLC1A1* and *SLIT3* on Mtb-induced cytokine induction. Three or more siRNA sequences per gene were used to select an optimal targeting siRNA resulting in a more than 70% decrease in target gene expression (Supplemental Figure 8). RNAi knockdown of *SLC1A1* significantly increased and *SLIT3* significantly decreased Mtb-induced IL1B protein secretion and mRNA expression robustly across multiple human donors and siRNA target sequence (Figure 4, A and B, and Supplemental Figure 9; *P* < 0.01). *SLIT3* knockdown also resulted in a highly significant decrease in *IL1B* expression in the media-only condition (Figure 4B; *P* < 0.0001). To assess a greater spectrum of inflammatory cytokines, we further assessed for the effect of *IL6* and *TNF* expression. *SLIT3* knockdown was associated with decreased Mtb-induced IL6 and TNF protein secretion (Figure 4A; *P* < 0.05) as well as mock-infected *TNF* mRNA expression (Figure 4B; *P* < 0.01). In summary, these in vitro data indicate that expression of the candidate genes *SLC1A1* and *SLIT3* is associated with Mtb-induced cytokine responses in macrophages.

### Exogenous application of N- and C-terminal human SLIT3 increases the Mtb-induced cytokine response.

We next assessed whether extracellular application of purified SLIT3 regulated cytokine induction. *SLIT3* is cleaved into an N-terminal and a C-terminal component, which bind to the ROBO and PLEXIN A receptors, respectively (61–64). Two of the four ROBO family receptors, *ROBO1* and *ROBO3*, as well as four PLEXIN A family receptors were found to be expressed in human monocytes (Supplemental Figure 10). When N- and C-terminal SLIT3 were coapplied, Mtb-induced IL1B and TNF protein secretion was significantly increased (Figure 4C; *P* < 0.05). For Mtb-induced TNF, a significant effect is seen with N-SLIT3 pretreatment alone, and this was amplified when both fragments were applied. In the uninfected conditions, SLIT3 pretreatment induced IL6 and TNF but not IL1B secretion, with an effect that was largest with cotreatment of both the N- and C-terminal fragments (Figure 4C; *P* < 0.0001). However, in contrast to Mtb-induced TNF, only the C-SLIT3 fragment led to a significant increase in IL6 secretion baseline, with the N-terminal having no significant effect. Taken together, these results indicate that receptors for both the N- and C-terminal of SLIT3 are likely to be present in human MDMs and that N- and C-terminal SLIT3 treatment creates distinct effects on the cytokine response, which are amplified when they are applied simultaneously.

### SLIT3 N- and C-terminal fragments induce increase in Mtb replication in MDMs.

**Figure 5:**
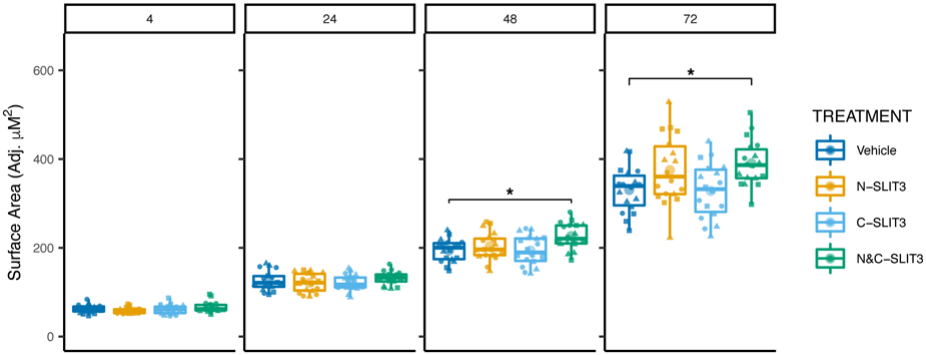
Evaluation of SNP-associated gene effect on other Mtb-response phenotypes reveals SLIT3 effect on intracellular Mtb. Intracellular Mtb replication in human macrophages assessed after hSLIT3 N- and C-terminal cotreatment using fluorescence microscopy to measure Mtb-mCherry surface area within each well over 72 hours. Four-hour time point quantifies differences in initial phagocytic uptake and shows no significant differences between groups. Results shown are from 6 biological replicates in 3 human donors. Significant differences were evaluated at each time point and show a significant increase in intracellular Mtb replication with cotreatment of N- and C-terminal SLIT3 at 48 and 72 hours. Significance was assessed using a linear mixed model adjusting for random effect of donor followed by ANOVA and pairwise comparisons for each time point. Individual data points were plotted according to model calculated values adjusted for donor intercept. *P < 0.05. Individual donor values are presented in Supplemental Figure 6E.

We next assessed whether *SLIT3* had an effect on other Mtb-response macrophage phenotypes, including phagocytosis and intracellular replication. Using a microscopy-based assay measuring surface area of fluorescently labeled Mtb, we quantified relative abundance of macrophage-associated Mtb at 4 hours after infection as well as growth over a 72-hour period. No significant differences in phagocytic uptake were seen between any SLIT3 treatment and vehicle-treated control after 4 hours (Figure 5). However, the Mtb fluorescence surface area was increased in the N- and C-terminal–cotreated samples at both the 48- and 72-hour time points (Figure 5; *P* < 0.05). The N-terminal–only pretreatment had a trending but non-significant effect on growth at 72 hours, whereas the C-terminal–only pretreatment had no significant effect on Mtb growth at any time point. Overall, these results reveal that coapplication of SLIT3 N- and C-terminal fragments has a significant effect on intracellular replication of Mtb that is not due to initial differences in phagocytic uptake.

### Assessment of candidate genomic loci mechanism of effect.

Since the candidate functional genes, *SLC1A1* and *SLIT3*, showed a validated effect on Mtb-induced cytokine induction, we further sought to identify the causal genetic mechanisms of their corresponding loci. The lead SNP of the *SLC1A1* locus, rs10974620, was previously identified as an eQTL in lymphoblastoid cell lines (*P* < 1 × 10^–5^) (65). Within the iMETHYL database, which assesses the immune cell methylation landscape, rs10974620 was located at a highly methylated site in CD14^+^ monocytes and had significant methylation quantitative trait loci (mQTL) associations with numerous adjacent probes (*P* < 1 × 10^–10^) (66). In the Uganda cohort, the uncommon allele of rs10974620 was associated with increased levels of *SLC1A1* expression in the Mtb but not the media condition (Supplemental Figure 11A; *P* < 0.01). To validate the iMETHYL findings, we used previously published global methylation profiling of CD14^+^ monocyte samples from our Ugandan cohort (67). Using the closest proximal methylation probe shared with the iMETHYL database, we validated a significant decrease in methylation according to rs10974620 allele frequency (Supplemental Figure 11B; *P* < 1 × 10^–8^).

For *SLIT3*, the lead SNP rs79380271 was not associated with eQTL effects. To determine whether there were protein quantitative trait loci (pQTL) effects, we also assessed secreted and cell lysate levels of SLIT3 protein abundance via ELISA, in MDMs from human donors in our Seattle cohort with various rs79380271 genotypes after 24 hours of media or TBWCL stimulation. Although we did not detect secreted SLIT3, we consistently identified SLIT3 protein levels in MDM lysate, which were decreased by TBWCL stimulation (Supplemental Figure 10C; *P* < 0.05). Interestingly, we observed a suggestive decrease in SLIT3 protein levels after media and TBWCL stimulation according to rs79380271 uncommon allele frequency in a small sample set (*n* = 10, *P* = 0.17 and 0.19, respectively) (Supplemental Figure 11C). Together, these data suggest that these genomic loci may regulate Mtb-induced *IL1B* expression through causally linked differences in levels of *SLC1A1* and *SLIT3*.

### Association of genetic variants with clinical TB phenotypes.

Since the Mtb-induced cytokine response plays diverse roles in modulating the spectrum of clinical TB progression, we next explored whether our *SLC1A1* and *SLIT3* loci were associated with clinical TB phenotypes. We assessed 4 clinical phenotypes ranging in severity, including tuberculin skin test/IFN-γ release assay (TST/IGRA) conversion, pulmonary TB (PTB), tuberculous meningitis (TBM), and TBM survival. Association with TST/IGRA conversion was tested using the *SLC1A1* lead SNP (rs10974620) and the *SLIT3* lead SNP (rs79380271) in a previously characterized Ugandan household contact cohort (9). PTB, TBM, and TBM survival association was similarly tested in a previously characterized Vietnam cohort (68–71). Owing to population differences, the rs79380271 genotype was not present in the Vietnam dataset, and the *SLIT3* Seattle lead SNP (rs62378623) was used as a proxy. Although no significant association was found for TST/IGRA conversion or PTB, both loci had SNPs associated with TBM phenotypes. The *SLC1A1* SNP, rs10974620, was associated with higher susceptibility to TBM (odds ratio, 1.238; 95% CI, 1.037–1.478; *P* = 0.018) (Table 2). Additionally, TBM mortality was higher among individuals with the *SLIT3* SNP, rs62378623, T/C genotype (6/9, 66.6%) versus the T/T genotype (57/360, 15.8%; hazard ratio, 23.63; 95% CI, 5.59–99.77; *P* = 1.05 × 10^–4^). Furthermore, evaluation of surrounding SNPs within each locus identified many SNPs in high LD that also showed significant association (Supplemental Figure 12). Together, these data indicate that the genomic loci within *SLC1A1* and *SLIT3* that are associated with Mtb-induced cytokine expression are also associated with the severe clinical TB phenotypes, TBM and TBM survival.

## Discussion

The major genetic variants regulating the macrophage cytokine response to live Mtb have not been defined. Using a cellular GWAS, we identified candidate genetic regulators of the macrophage Mtb response, validated several of these regulators in vitro, and linked them to severe clinical phenotypes. We identified 77 candidate genomic loci with a suggestive association with Mtb-induced cytokine expression. We further explored and genetically validated lead candidate SNPs in a second population. Follow-up macrophage assays confirmed Mtb-induced cytokine regulation by PLA2, *SLC1A1*, and *SLIT3*. SLIT3 stimulation was also found to augment Mtb replication in macrophages. The lead *SLC1A1* and *SLIT3* variants were identified as possibly regulating corresponding levels of their associated mRNA and protein abundance, respectively. In addition, *SLC1A1* and *SLIT3* variants were significantly associated with TBM phenotypes in Vietnam. Our findings support the use of a cellular GWAS to identify host regulators of TB pathogenesis with clinical consequences.

Although no variants passed a traditional significance threshold in the Uganda discovery cohort (*P* < 5 × 10^–8^), in-depth annotation of each suggestively associated locus (*P* < 1 × 10^–5^) highlighted many loci of interest, which could serve as a potential hypothesis-generating resource for further in vitro validation. Multiple identified loci were mapped to genes previously associated with regulation of cytokine levels and response pathways. Two of these genes, *NEDD4* and *IFNAR2*, have already been shown to affect intracellular replication of Mtb (52, 53). This is consistent with previous cellular GWAS, which have often been able to identify multiple important genetic insights controlling a phenotype within a single study (22, 23, 25–28). Many lead SNPs had shared association with basal media cytokine expression, which may suggest that loci effects are not restricted to Mtb induction and may have broader inflammatory implications. Lead SNPs for 26 of the 77 identified loci were found to be at least 10 times more common in the AFR panel of the 1000 Genomes Project than in any other 1000 Genomes panel (American, East Asian, European, or South Asian; Supplemental Table 2). This suggests that many of these variants that were specifically identified using a cohort of Ugandan individuals may not have been discovered if analyses were performed in other populations. This is similar to prior observations in other cellular GWAS and highlights the importance of evaluating populations that are often underrepresented in genetic studies (22).

In addition to annotation of individual genetic variants, we also performed gene and gene set analysis to determine whether a joint effect of multiple SNPs was enriched in a biologically relevant way. SNPs associated with Mtb-induced *TNF* expression were enriched in two separate canonical pathways. To our knowledge, this is one of the first times that genetic regulators of cytokine induction have been identified using the aggregate effect of genome-wide SNPs. One of the two pathways identified was in α-linolenic acid metabolism, which was previously implicated in numerous facets of the host response to Mtb, including cytokine induction, cell death, and effect on intracellular replication (57–59). Much of the signal within the pathway was concentrated among a variety of unrelated PLA2 isoforms, which serve as an important source of arachidonic acid (AA) by catalyzing its release from membrane phospholipids (60). AA and its downstream intermediates have diverse pro- and antiinflammatory effects on the host response to Mtb that can vary based on cell type, host species, and mycobacterial strain (72–75). Interestingly, we found that PLA2 inhibition altered TNF levels in opposite directions when comparing TB whole-cell lysate and live Mtb. The substantial difference in lysate versus live Mtb may be in line with prior research in which H37Rv was able to modulate the products of AA metabolism differently from the avirulent strain H37Ra so that the antiinflammatory lipoxin LXA_4_ was favored over the inflammatory prostanoid PGE_2_ (76). Regulation of lipoxin levels has previously been shown to be essential in Mtb response, and *LTA4H* mutations that lead to increases in LXA_4_ have previously been shown to affect TNF production, and to have clinical associations with PTB, multibacillary leprosy, and TBM (77). In addition, some of the genetic associations may extend to treatment responses to steroids and highlight potential clinical applications (78). Although further research is necessary to ascertain the exact mechanism of effect, these data highlight the large and complex role that PLA2 inhibition plays in the human macrophage cytokine response and suggest that genetic variants causing differences in PLA2 activity could play a substantial role in controlling the extent of an individual’s Mtb-induced cytokine response.

In order to characterize SNPs with a more global impact, we examined an independent Seattle population cohort and identified 4 loci associated with Mtb-induced cytokine expression with consistent directionality of effect in both populations. The effect on Mtb-induced *IL1B* levels was validated for 2 of these loci, *SLC1A1* and *SLIT3*, with *SLIT3* showing an additional effect on IL6 and TNF secretion. *SLIT3* has been implicated in cytokine regulation previously, showing a similar proinflammatory effect in human gestational tissue (47). However, another SLIT family member, *SLIT2*, has also been implicated in antiinflammatory actions through its interactions with the *ROBO4* receptor in endothelial cells (79). This suggests that SLIT family members may have different functions in the cell or that a combination of receptors present on each cell type can result in a distinct response. We detected expression of *ROBO1* and *ROBO3* in monocytes, while *ROBO4* was absent. As such, SLIT3-ROBO interaction could have a distinctly proinflammatory effect in human macrophages. Although the SLIT3 N-terminal fragment interaction with the ROBO receptor has been previously described, the C-terminal fragment was more recently discovered to bind the PLEXIN A receptor family (64). *PLEXINA4* has previously been implicated in regulation of TLR-induced cytokine signaling in myeloid cells, and in a prior cellular GWAS association of *TNF* response to Mtb purified protein derivative (25, 80). To our knowledge, this is the first time that *SLIT3* regulation of the cytokine response in human macrophages has been investigated, and the first identification of simultaneous effects of SLIT3 N- and C-terminal on cytokine production. *ROBO1* receptor binding is largely thought to act through downstream RHOA GTPase activation, whereas PLEXIN A receptor binding often acts through RAC1 GTPase activation (63, 80). Although the mechanistic details have not been clarified in this study, it is intriguing to postulate that by targeting both of these pathways simultaneously, *SLIT3* can exert an increased response, further adding to the multi-faceted effect that SLIT3 could have on a cell-to-cell basis depending on the receptors it expresses. We additionally identified that *SLIT3* was also associated with increasing intracellular Mtb replication. While mechanistically investigating the *SLIT3* locus, we identified rs79380271 as a potential pQTL associated with trending decreases in SLIT3 protein abundance. Although a larger sample size is needed, this would match a model in which genetically associated decreases in levels of SLIT3 protein result in decreased Mtb-induced *IL1B*. The directionality of this correlation is supported both in the *SLIT3* siRNA data, where knockdown decreased cytokine levels, and during overexpression via exogenous SLIT3 treatment, which increased cytokine levels. Interestingly, *SLIT2* was recently found to be actively upregulated during Mtb infection and led to differences in host oxidative responses (81). In contrast, we found that *SLIT3* expression and protein abundance were extensively downregulated during Mtb and TBWCL stimulation, respectively (Supplemental Figure 10, B and C). Lastly, we identified a genetic link between *SLIT3* variants that regulate the Mtb-induced cytokine response and differences in TBM survival. In addition to the currently identified role in cytokine regulation, *SLIT3* has also been implicated in modulating monocyte chemotaxis (63). As such, *SLIT3* could modulate TBM survival through differential cytokine response, control of Mtb replication, immune cell recruitment, or a combination of the three. Further elucidation of this mechanism may illuminate whether the association of *SLIT3* genetic effects on TBM survival modulates the effects of steroids and/or LTA4H-dependent pathways. For example, an LTA4H genetic variant was associated with cerebrospinal fluid cytokines and inflammatory cell recruitment, a mechanism that could also be impacted by *SLIT3* and contribute additional effects on survival (78, 82).

We additionally identified *SLC1A1* as a major regulator of Mtb-induced *IL1B* levels. *SLC1A1* is a glutamate and aspartate transporter and modulates glutamate levels in neuronal tissue. Amino acid transporters have been reported to have major effects on cytokine production, and previous cytokine cellular GWAS studies identified an amino acid transporter, *SLC36A4*, in *Staphylococcus aureus*–induced IL-22 levels (23). Modulation of aspartate transport and glutaminolysis through glutamate transport has previously been implicated to have major effects on the macrophage response (83, 84). Mice with heterozygous *SLC1A1* deletion have different cytokine profiles and neuroinflammation compared with wild type (48). The lead SNP identified in our study, rs10974620, has been identified in previous genetic studies to have a significant association with post-traumatic seizure (85). rs10974620 was identified to have previous eQTL associations in the literature, and we validated that it was associated with significantly increased Mtb-induced expression of *SLC1A1* in the Ugandan cohort (65). Furthermore, rs10974620 was identified to directly modify a highly methylated site in CD14^+^ monocytes (66), and was associated with significant decreases in methylation of numerous adjacent probes. We further confirmed rs10974620 mQTL status for one of these within CD14^+^ monocytes of the Ugandan cohort. Interestingly, if the uncommon allele of rs10974620 is associated with increased *SLC1A1* and decreased Mtb-induced *IL1B*, this would match the directionality of our siRNA data in which *SLC1A1* knockdown resulted in increased Mtb-induced *IL1B*. We also found that rs10974620 was associated with susceptibility to TBM in a Vietnam cohort. Given *SLC1A1*’s effect on neuroinflammation in mice, it is possible that *SLC1A1* regulation of the cytokine response could play a substantial role in meningeal inflammation in TBM. Importantly, the identification of significant clinical associations with variants identified in this cellular GWAS approach highlights its ability to lead to variants with clinical impact.

Our study has several limitations. First, although significant genetic effect was found at the gene set level, no individual variants in the Ugandan cohort passed a traditional genome-wide significance threshold of *P* < 5 × 10^–8^. An increased sample size with our discovery cohort could address this limitation. We mitigated this limitation by using an independent cohort to validate population spanning effects. In addition, key findings were validated with in vitro studies. Second, since our aim was to capture the early Mtb-induced myeloid cell response, we chose to only focus on monocyte expression of 4 cytokines at an early 6-hour time point for our initial GWAS. Future studies could benefit from additional cytokines, time points, cell types, and assessment of cytokine protein levels. Lastly, although multiple genetic regulators of Mtb-induced cytokine expression were identified and had a validated effect in vitro, their genetic mechanism of effect remains to be fully defined. Although we believe that the possible eQTL and pQTL associations that match in vitro observations are promising, we have not identified the causal mechanisms leading to their QTL status. Further studies confirming these associations and elucidating the mechanisms by which they have an effect are of crucial importance to fully define the genetic regulators of the Mtb-induced cytokine response and will be the aim of future research.

In summary, we used a cellular GWAS approach to characterize the major genetic regulators of the Mtb-induced cytokine response in myeloid cells and identified candidate genetic loci, pathways, and genes that may have an important role in Mtb-response biology. Further defining the mechanisms by which these genetically regulated genes control Mtb-induced cytokine infection could yield important insights in our understanding of differences in host disease progression and increase our ability to respond to the global burden of TB.

## Methods

### Sex as a biological variable.

Our study examined 3 human cohorts that all contained both males and females (Supplemental Table 1). Sex was included as a covariate in all discovery and validation genetic analyses to control for differences that may be specific to one sex.

### Human donor and clinical cohorts.

Our GWAS involved ex vivo characterization of monocytes of human donors from Kampala, Uganda. Individuals from Uganda were part of a highly Mtb-exposed Ugandan household contact cohort. Subjects were recruited between 2002 and 2012 and followed for serial TST and IGRA testing over an 8- to 10-year period, after which peripheral blood mononuclear cells (PBMCs) were collected. Our study included RNA-Seq characterization of 100 individuals within this cohort who either were resistant to TST/IGRA conversion (RSTR) or converted to latent TB infection (LTBI) as described previously (41). Mtb-induced cytokine GWAS was performed on both RSTR and LTBI individuals without clinical phenotype stratification. Assessment of clinical differences in TST/IGRA conversion was performed using a larger 325-individual cohort (97 RSTR, 228 LTBI) previously described (9). Our independent Seattle cohort analysis involved healthy donors from Seattle who were recruited between 2020 and 2022, and PBMCs were collected at the time of recruitment. Individuals who reported no current or previous history of serious medical health issues were included. Subjects of the Vietnam clinical cohort were collected over several sites in Ho Chi Minh City and were enrolled with either PTB or TBM, and individuals with TBM were followed for survival outcome (68–71). TBM treatment often consisted of dexamethasone coadministration. We compared genotype frequencies of SNPs in PTB and TBM patients compared with control individuals within the Vietnam population, who have been previously described (86).

Details, including clinical definitions, are published for the Ugandan and Vietnam cohorts (9, 68–71), and clinical cohort tables are included (Supplemental Table 1).

### Cell culture, reagents, and CD14^+^ isolation.

Human monocytes and macrophages were cultured in RPMI 1640 (Gibco) supplemented with FBS (Atlas Biologicals) at a final concentration of 10% and macrophage colony-stimulating factor (M-CSF; PeproTech) at a final concentration of 50 ng/mL for all experiments. Monocyte preparation from the Uganda cohort was previously described (41). In brief, PBMCs were thawed and rested for 1 day before use of negative CD14 isolation (Miltenyi, Monocyte Isolation Kit II) to enrich for monocytes, which were plated and rested for an additional day before infection. For the Seattle healthy donors, macrophages were obtained from cryopreserved PBMCs, which were thawed, resuspended at 2 million cells/mL, and differentiated for 5 days in indicated culture media with M-CSF. Monocyte-derived macrophages were then isolated using magnetic bead column purification via negative selection (Miltenyi, Pan Monocyte Isolation Kit).

### Mtb-induced cytokine expression and secretion.

Monocytes and macrophages were infected with H37Rv Mtb thawed from cultures previously grown to mid log phase and washed twice in Sauton’s medium, at a multiplicity of infection (MOI) of 1, or subjected to a mock infection media condition. For Mtb-induced cytokine expression, cells were lysed in Trizol after 6 hours, after which RNA was isolated using a modified miRNeasy protocol (QIAGEN). RNA processing was performed as previously indicated for the Uganda cohort (41). Briefly, RNA was submitted for RNA-Seq in 2 batches, followed by subsequent ComBat-seq processing to correct batch effects (87). Merged RNA-Seq counts were then converted to log_2_ counts per million (CPM) using voom, and Mtb-induced cytokine expression of *IL1B*, *IL6*, *TNF*, and *IFNB1* was determined by change in log_2_ CPM between Mtb and media conditions within each individual (88). RNA processing for the Seattle cohort was performed by cDNA synthesis and assessed via reverse transcriptase PCR normalized to GAPDH control with subsequent log fold change determination between Mtb-induced and media samples. Cytokine quantitative PCR assays were purchased from Integrated DNA Technologies (IDT) (IL1B, Hs.PT.58.1518186; IL6, Hs.PT.58.40226675; TNF, Hs.PT.42.1656119; and IFNB1, Hs.PT.58.39481063.g) and endogenous control GAPDH from Applied Biosystems (4310884E). Cytokine secretion was characterized using similar infection conditions for 24 hours, after which supernatants were extracted, filtered, and analyzed via ELISA for IL1B, TNF, and IL6 (R&D Systems, Duoset).

### Genotyping and imputation.

For the Uganda cohort, genotyping was performed using MEGA^EX^ and OMNI5 genotyping chips (Illumina) as previously indicated (9, 89). SNPs with greater than 99% call rate, 1 × 10^–6^ Hardy-Weinberg equilibrium (HWE), and 1% minor allele frequency (MAF) were subsequently aligned to a reference genome according to TOPMed Freeze 8 variants and imputed using the TOPMed server (90). Overlapping SNPs within both imputations were combined and filtered for imputation quality score greater than 0.5, greater than 5% MAF, and greater than 1 × 10^–6^ HWE before testing. The Seattle cohort was genotyped using a MEGA^EX^ Illumina panel, and subjected to similar initial pre-imputation quality control, mapped to a Human Resource Compendium (HRC) variant in the reference genome, and imputed using the Michigan Imputation Server (https://imputationserver.sph.umich.edu/) and an HRC reference panel (91). SNPs were subsequently filtered for greater than 5% MAF, greater than 1 × 10^–6^ HWE, for overlapping SNP analyses.

### GWAS analysis and population spanning cohort analysis.

The cellular GWAS in the Uganda cohort was performed using the GENESIS package in R (92). We used a linear model adjusted for age, sex, experiment (RNA-Seq batch), genotypic PC1, genotypic PC2, and kinship to examine whether SNPs were associated with Mtb-induced cytokine expression (Mtb media value). Covariates were included in final model analysis if found to significantly affect the distribution of any of the 4 Mtb-induced cytokines assessed using a linear model (Supplemental Figure 1A). The number of PCs included in the analysis was determined using an elbow plot to select the number of PCs at which variation with additional PCs added was minimal. Genotyped SNPs were used to generate PCs and kinship matrix using KING kinship estimation (https://www.kingrelatedness.com/manual.shtml). The genomic inflation factor was assessed for each cytokine (Supplemental Figure 1A). Population spanning significance in the Seattle cohort was similarly tested in a genome-wide fashion using the same workflow, and only specifically queried SNPs were extracted for analysis.

### Functional annotation of genomic loci and eQTL analysis.

Individual SNP results were clustered into genomic loci using a PLINK clustering protocol in which SNP summary *P* values were used to group each locus with an SNP surpassing *P* < 1 × 10^–5^ into a lead SNP and group all less significant linked SNPs within PLINK default 250 kb, at an LD threshold of 0.1 (93). All SNPs surpassing *P* < 1 × 10^–3^ within each locus were then annotated using Ensembl Variant Effect Predictor for proximal gene mapping, CADD score, and global MAFs (43). Assessment of eQTL effect was similarly performed using all SNPs surpassing *P* < 1 × 10^–3^ tested for associated differences in *cis* gene media expression (+/– 250 kb) using a linear mixed model in the KIMMA package adjusting for sex, age, batch, PC1, PC2, and kinship (94). Similar assessment was performed for the single query of Mtb-induced *SLC1A1* expression for rs10974620. All SNPs within each locus with *P* < 0.01 were additionally annotated for previously reported associations in the GWAS catalog to account for potential population differences in LD patterns (44). The rs10974620 and rs79380271 SNPs of interest were directly queried in the eQTL catalog and iMETHYL (65, 66). Lastly, SNPs of interest were visualized for global MAFs using the Geography of Genetic Variants browser (95).

### MAGMA gene set analysis.

Multi-marker Analysis of GenoMic Annotation (MAGMA) v1.07 was used to map SNP locations to strict gene coordinates with 0 kb flanks (54). Gene level *P* values were calculated using previously calculated GWAS-level *P* values for each cytokine with a multi=all model which aggregates the results of PC regression, a SNP mean model weighted towards detecting overall mean effect, and a SNP top model which is weighted towards detecting the effect of small proportions of variants. C2-canonical pathways (subset to KEGG, Reactome, Pathway Interaction Database, and BioCarta) and C5-GO gene sets were obtained from MSigDB (56). Gene sets with greater than 10 genes with gene-level *P* values were evaluated for significantly enriched signal after adjusting for multiple correction using default MAGMA settings. Gene sets were further analyzed for single-gene biasing of significance via early QQ plot deviation. Lastly, network interactions were assessed using STRING and visualized using Cytoscape (96, 97).

### PLA2 inhibitor assay.

AACOCF3 (Tocris Biosciences) was purchased and applied extracellularly to human MDMs as previously indicated for a 3-hour pretreatment in comparison with an EtOH vehicle control. TB whole-cell lysate (25 μg/mL; BEI Resources) or live Mtb infection as described above was used for 24 hours, and then supernatants were extracted and assessed for TNF, IL1B, and IL6 secretion using ELISA (R&D Systems, Duoset).

### siRNA knockdown in human macrophages.

siRNA knockdown was performed in human macrophages isolated after 5 days of differentiation in indicated culture media. Lonza P3 Primary Cell Nucleofector kit was used for nucleofections of 700,000 cells using 30 pmol of siRNA. Cells were rested for 48 hours before experimentation. Three separate siRNAs were tested for each gene, and the siRNA with the greatest knockdown efficacy of at least 70% in comparison with negative control siRNA was used for future experiments (Supplemental Figure 3). DsiRNA sequences were purchased from IDT for *SLIT3* primary (hs.Ri.SLIT3.13.1) and validation (hs.Ri.SLIT3.13.7) as well as for *SLC1A1* primary (hs.Ri.SLC1A1.13.1) and validation (hs.Ri.SLC1A1.13.2) and assessed using IDT quantitative PCR assays for *SLIT3* (Hs.PT.58.25920874) and *SLC1A1* (Hs.PT.58.19942121). All siRNA knockdown was performed in comparison with NC-1 negative control siRNA (IDT, 51-01-14-03), in 3 separate infections, using 3 distinct human donors.

### hSLIT3 N- and C-terminal treatments.

Recombinant human SLIT3 — N-terminal fragment AA 34-1116 (9255-SL) and C-terminal fragment (9067-SL) — was purchased from R&D Systems. Cell fragments were resuspended according to manufacturer protocol and kept frozen at –20°C until application, thawed, and resuspended in RPMI with 10% FBS. Cell fragments were applied 30 minutes before infection with Mtb as compared with a vehicle control with equal volume resuspension buffer (DPBS, Gibco). Similar SLIT3 fragment treatment was performed after the wash step of the initial 4-hour infection for intracellular replication assay.

### Intracellular replication of Mtb in infected macrophages.

H37Rv Mtb expressing mCherry was grown to mid log phase (optical density < 0.6), single-cell-suspended using filtration through a 5 μM filter, and diluted in RPMI to give final infection MOI of 1. Human macrophages were cultured in previously indicated media and rested 24 hours after column isolation. Infection was performed for 4 hours, after which supernatant was removed and cells were washed in HBSS 2 times before addition of fresh culture media. Intracellular replication was measured using total surface area of mCherry signal within a 96-well plate (Biotek Cytation 5, Agilent). Six total infections per condition per individual were measured. Any initial differences in phagocytosis were similarly determined by surface area at this 4-hour time point.

### Methylation assessment.

mQTL status was assessed in 40 individuals within the Ugandan cohort, who were simultaneously profiled in a recently published and described methylation dataset (67). Briefly, donor cryopreserved PBMC samples were thawed and rested 24 hours in above-indicated culture media followed by subsequent CD14^+^ cell isolation (Pan Monocyte Isolation Kit, Miltenyi). Cells were then replated and rested for an additional 24 hours and lysed in unstimulated state to extract genomic DNA using a Quick-gDNA Miniprep kit and bisulfate-treated with the EZ-96 methylation kit (Zymo Research). Converted DNA was assessed with the Infinium MethylationEPIC 850 BeadChip (Illumina) and sequenced. Poor-quality probes were filtered using ChAMP (98), and normalized β values were determined. The closest proximal probe with indicated significant β value differences in iMETHYL was identified at chr9:4559892. The cohort data for this probe were assessed for methylation differences according to rs10974620 allele frequency using a linear model in KIMMA adjusted for previously indicated covariates and kinship.

### SLIT3 protein abundance assessment.

Assessment of supernatant and lysate SLIT3 protein abundance was performed using a human SLIT3 protein ELISA kit (CUSABIO). Assays were performed using macrophages from 10 Seattle healthy donors. After 24 hours of TB whole-cell lysate (25 μg/mL; BEI Resources) or media-only treatment, supernatants were extracted and samples were lysed using Cytobuster Protein Extraction Reagent (Millipore). Protein abundance was assessed relative to a standard curve, and pQTL effects were assessed using a simple unadjusted linear model because of sample size limitations.

### Vietnam clinical phenotype association assessment.

We further evaluated the relevance of our candidate SNPs to PTB/TBM susceptibility as well as TBM survival in the Vietnamese population. Single-variant association analyses for susceptibility to PTB (1,598 cases and 1,267 controls) and TBM (407 cases and 1,139 controls) were performed using the R package SAIGE (v1.3.0) (99), with adjustments for the first 3 PCs to correct for population stratification. Effect size was estimated through Firth’s bias-reduced logistic regression, and odds ratio with 95% confidence interval (CI) was calculated based on effect size and its standard error of the mean. For TBM survival, analysis was performed via SPACox (100), which uses a saddlepoint approximation (SPA) to calibrate the test statistics and accurately control type I error rates. The first 3 genotypic PCs, age, and sex were included in the model as covariates. *P* value and hazard ratios corresponding to the score statistics were reported to reveal the significance and direction of the association.

### Statistics.

Genetic tests were performed as previously indicated using the GENESIS R package, assocTestSingle, accounting for necessary covariates, and genetic relatedness as a polygenic random effect (92). GENESIS uses a penalized quasi-likelihood approximation of the generalized linear mixed model as previously developed in GMMAT. A commonly used threshold *P* value of 1 × 10^–5^ was used for determining variants with suggestive association. Significance testing in the Seattle cohort was performed in a similar fashion initially only testing for lead SNPs evaluated for 1-way significance to account for directionality across populations at a nominal *P* < 0.05. The strict multiple correction threshold was determined by Bonferroni correction of this nominal *P* value by number of lead SNPs tested within each cytokine. Our Vietnam clinical query of PTB/TBM and TBM survival was performed in SAIGE and SPACox, respectively (99, 100). Our *cis*-eQTL analysis was performed using a linear model of each loci SNP association with differences in mock-infected RNA-Seq gene expression values located within +/– 250 kb of the SNP. Analysis was performed in KIMMA to adjust for covariates and kinship in a fashion resembling GENESIS (94). Final eQTL results were FDR-adjusted across all results. In vitro assay significance was assessed using a linear mixed model incorporating donor as a random effect. Differences in AACOCF3, siRNA, and SLIT3 treatment were then assessed for significant group differences via 2-way ANOVA, and pairwise comparisons were made and adjusted for multiple comparisons using Tukey’s method.

### Study approval.

All human-subjects work was approved by IRBs for the respective sites; Uganda-CWRU Research Collaboration (UCRC) in Kampala, Oxford University Clinical Research Unit in Ho Chi Minh City in Vietnam, and the University of Washington in Seattle.

### Data availability.

Supporting data values for all figures are provided in the Supporting Data Values file. Access to raw transcriptomic data for the Ugandan cohort is available through the NCBI database of Genotypes and Phenotypes (dbGaP) Data Browser using accession number phs002445.v1.p1, but requires approval by data access committees for each study site (41). Mtb-induced cytokine GWAS summary statistics for the Uganda and Seattle cohorts for all significant results (*P* < 0.05) and R code is available at https://github.com/hawn-lab/TBCytoGWAS_public/ Github commit ID 1e8b254 and URL https://github.com/hawn-lab/TBcytoGWAS_public/commit/1e8b254f247122276921d9ae1295b75fe856787a

## Author contributions

All experiments were performed by JJI, JDS, GJP, MA, and LEV with input on design from TRH. Computational analyses were performed by JJI and KADM. Statistical genetic analyses were performed by PHB and CMS for the Ugandan cohort and by XC and SJD for the Vietnam cohort. The manuscript was written by JJI and TRH. All authors reviewed the manuscript. Clinical cohorts were established and characterized by CMS, HMK, and WHB for the Ugandan cohort and by DTMH, HDTN, CCK, GET, HTH, NTTT, and SJD for the Vietnamese cohorts.

## Supplementary Material

Supplemental data

Supplemental table 1

Supplemental table 2

Supplemental table 3

Supporting data values

## Figures and Tables

**Table 1:**
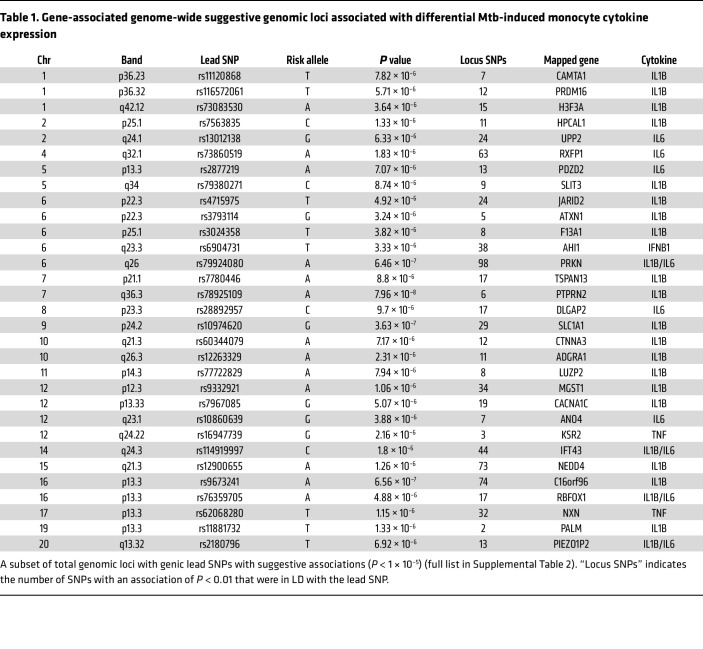
Gene-associated genome-wide suggestive genomic loci associated with differential Mtb-induced monocyte cytokine expression

**Table 2:**
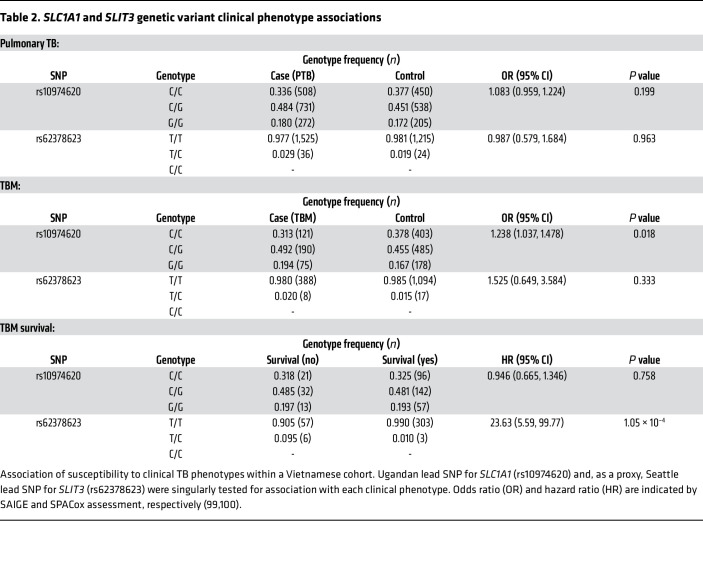
*SLC1A1* and *SLIT3* genetic variant clinical phenotype associations

## Version 1 05/22/2025

In-Press Preview

## Version 2 07/15/2025

Electronic publication
