# Supplementary material for: Myeloid cell genome-wide screen identifies variants associated with Mycobacterium tuberculosis–induced cytokine transcriptional responses
Source: J Clin Invest. 2025 May 22;135(14):e179822. doi: 10.1172/JCI179822 (PMC12259255; doi:10.1172/JCI179822)
Supplement: Supplemental data [file jci-135-179822-s089.pdf]

Supplemental Materials:

Supplemental Figures:

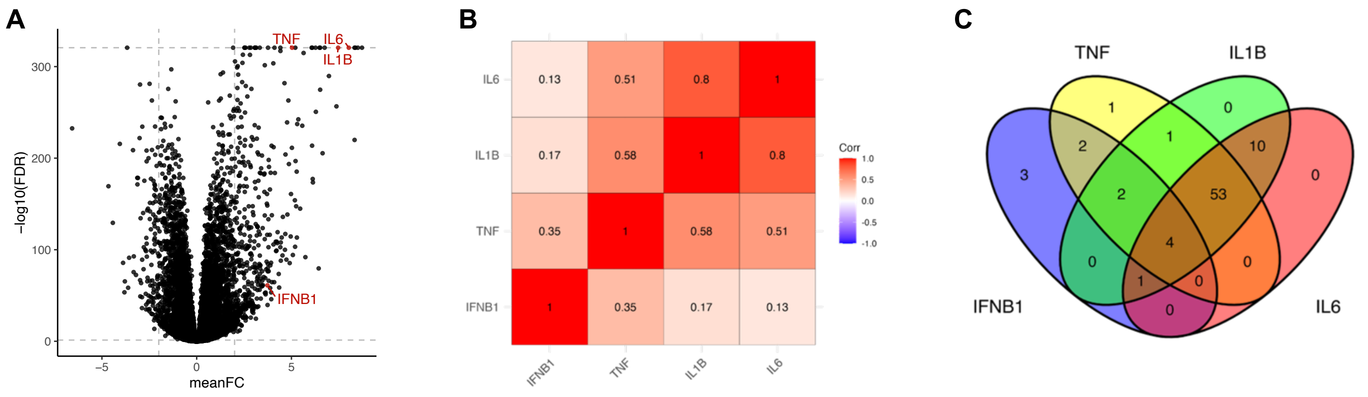

Supplemental Figure 1: Mtb-induced cytokine expression, correlation, and shared SNP and cytokine expression associations. (A) Volcano plot of mean fold change in gene expression in 6hr Mtb compared to Media stimulated monocytes via RNAseq assessed with a linear mixed model in the Ugandan cohort with highly induced cytokines highlighted (B) The correlation of all four TB-media cytokine expression values with each other shows a high correlation among inflammatory cytokines *IL1B*, *IL6*, and *TNF*, and a minor correlation between *TNF* and *IFNB1*. (C) Shared SNP and cytokine expression associations was assessed for each of the 77 suggestive loci at a nominal, $P < 0.05$ threshold and showed a majority (53/77) of the loci have some association in *IL1B*, *IL6*, and *TNF* simultaneously.

A

Covariate	IL1B	IL6	TNF	IFNB1
AGE	0.1927	0.0460*	0.00748**	0.7672
SEX	0.7049	0.6940	0.1263	0.7757
Experiment	0.9407	0.8971	0.1831	0.0001***
Sample Group	0.2160	0.3167	0.4144	0.2140
**Final Inflation factor (λ)**	**1.0371**	**1.0163**	**0.9866**	**0.9319**

B

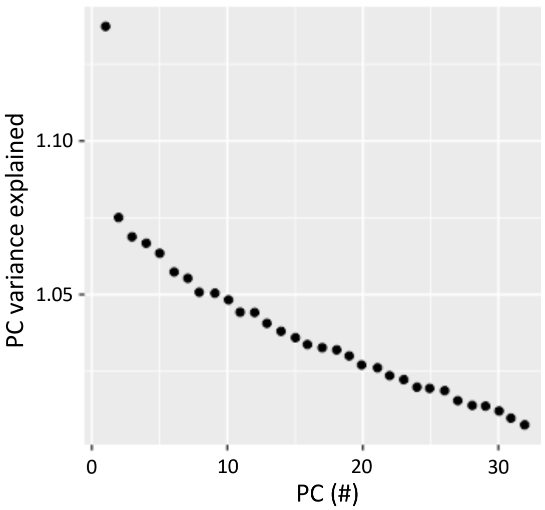

Supplemental Figure 2: Results of covariate inclusion analyses and final effect on lambda inflation factor. (A) Significance of differences in TB-Media cytokine expression distribution according to each of the covariates, age, sex, RNAseq batch (experiment), and LTBI or RSTR clinical status (sample group) using a linear model. Significance determined as (* $P < 0.05$, ** $P < 0.01$, *** $P < 0.001$). Final lambda scores indicating inflation of GWAS results shown for each cytokine showing negligible inflation of results. (B) Elbow plot of variance explained for each genotypic pc showing little additional variability added with each pc past pc2.

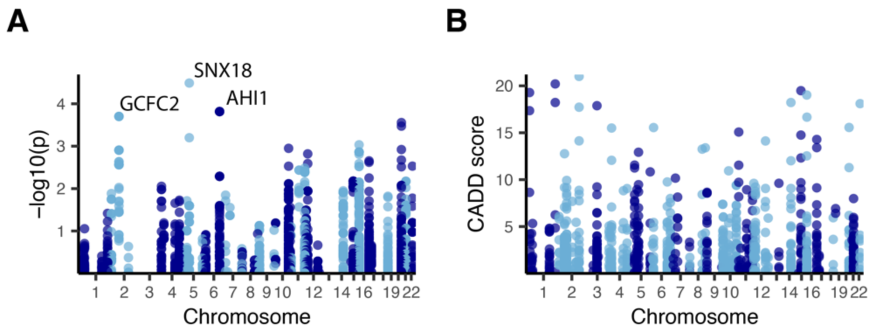

Supplemental Figure 3: Genome-wide locus eQTL and CADD score plots. Clumped loci SNPs in linkage with lead SNPs that have significance $P < 0.001$ were assessed for (A) cis-eQTL associations and (B) annotated for CADD scores. Results show many loci have numerous SNPs showing evidence of cis-eQTL signals and impactful CADD scores. Cis-eQTL analysis performed on media expression of genes within 250kb of indicated SNPs using mixed linear model adjusting for genotypic pc1, pc2, sex, age, experiment, and kinship. Significant eQTLs after FDR multiple correction are indicated with labeled effected genes.

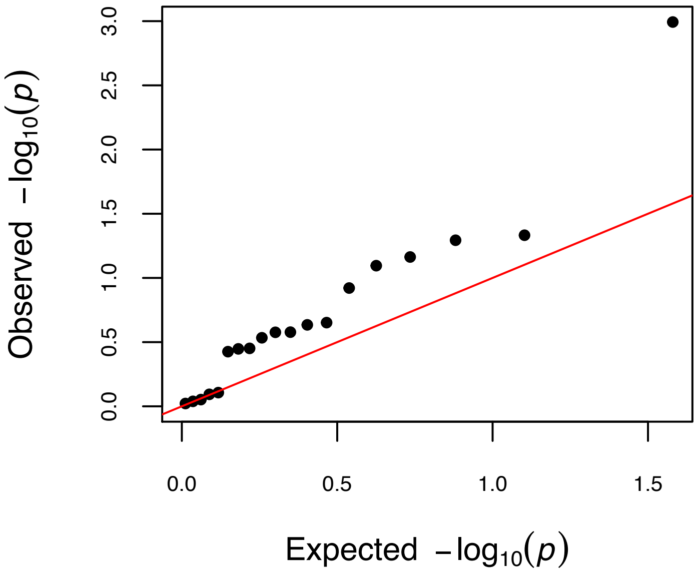

Supplemental Figure 4: QQ plot of gene *P*-values of the alpha-linolenic acid metabolism gene set. MAGMA calculated gene *P*-values for association with Mtb-induced *TNF* expression were plotted for the 19 genes in the alpha-linolenic acid metabolism pathway and show an early deviation from expected trajectory suggesting a gene set rather than single gene effect.

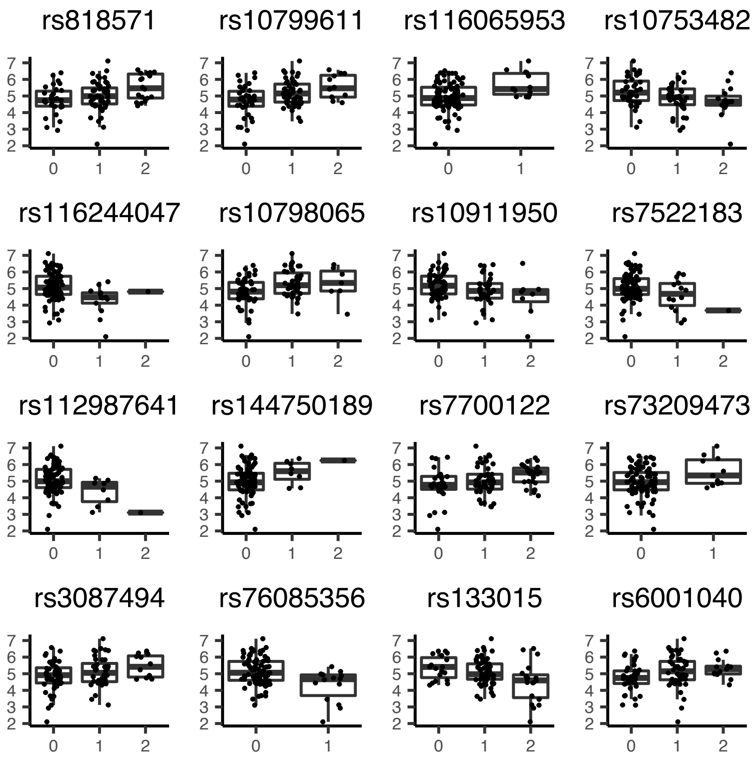

Supplemental Figure 5: Genotypic mean Mtb-induced *TNF* expression plots of lead SNPs across gene set. 16 Independent lead SNPs which contributed to the top 6 gene level significances were plotted for association with Mtb-induced *TNF* in the Uganda cohort. Despite highly variable allele frequencies and directions of effect, all SNPs plotted show effects on Mtb-induced *TNF* expression and help to explain significance of overall results.

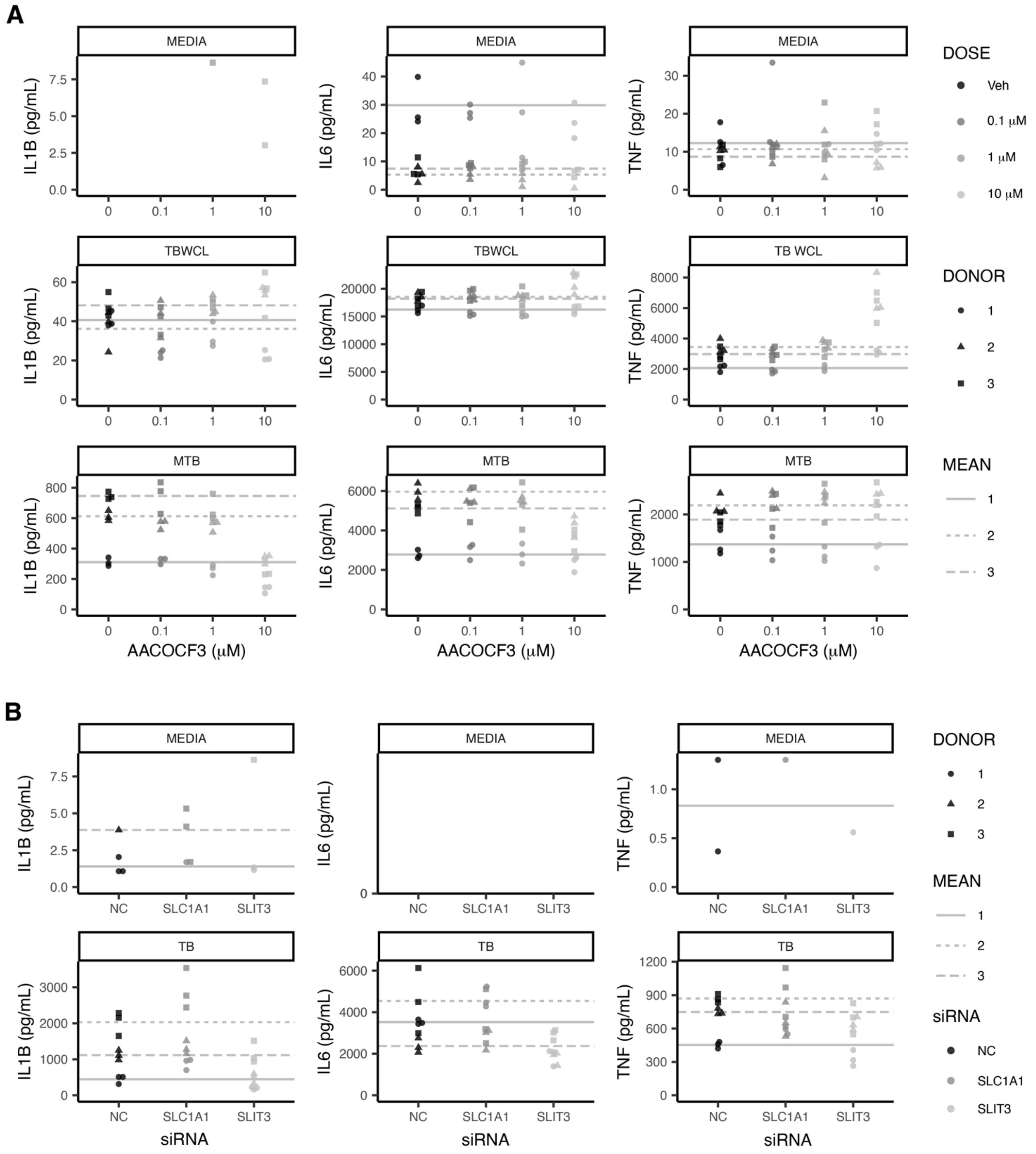

A
MEDIA
TBWCL
TB WCL
MTB
IL1B (pg/mL)
IL6 (pg/mL)
TNF (pg/mL)
AACOCF3 (μM)
0
0.1
1
10
DOSE
Veh
0.1 μM
1 μM
10 μM
DONOR
1
2
3
MEAN
1
2
3
B
MEDIA
TB
IL1B (pg/mL)
IL6 (pg/mL)
TNF (pg/mL)
NC
SLC1A1
SLIT3
siRNA
DONOR
1
2
3
MEAN
1
2
3
siRNA
NC
SLC1A1
SLIT3

C

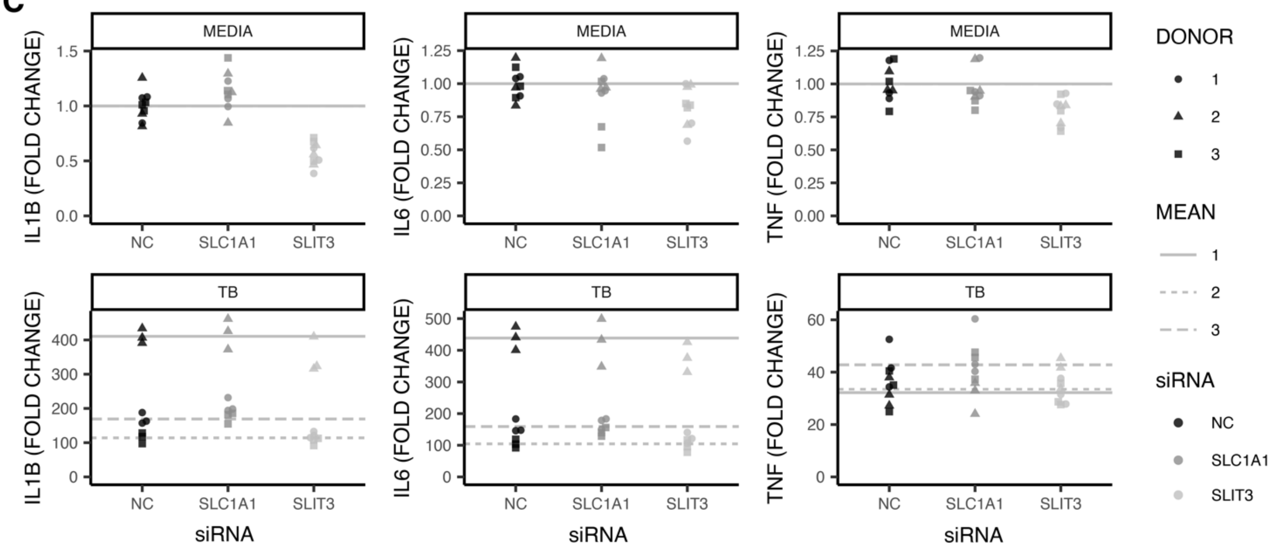

D

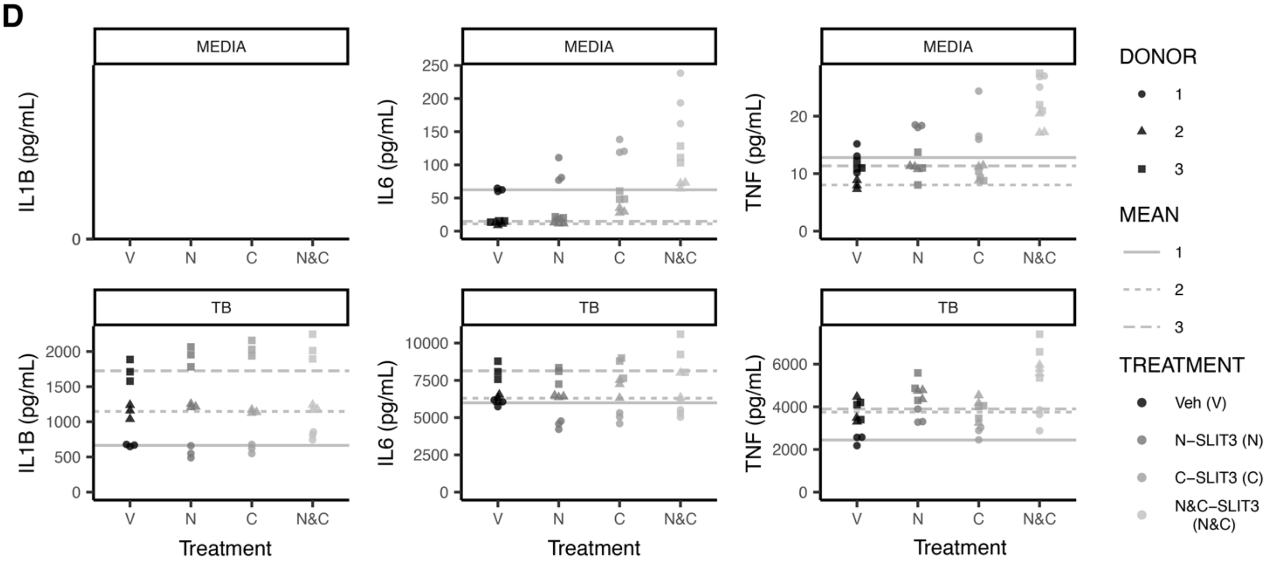

E

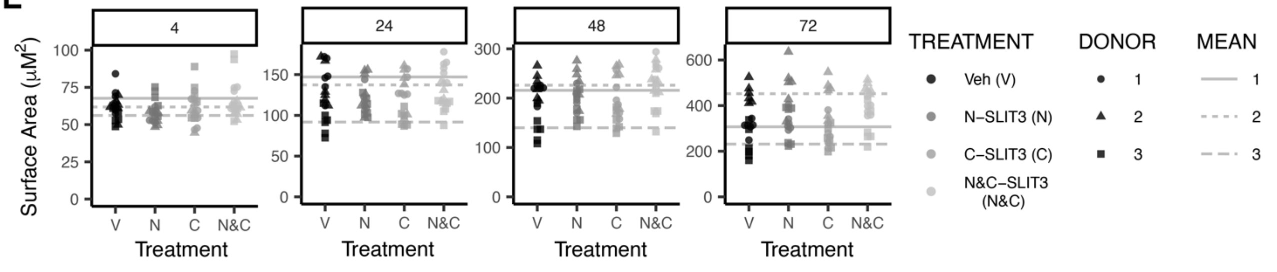

Supplemental Figure 6: Unadjusted results of *in vitro* experiments. Results for each *in vitro* experiment are plotted without adjustment for donor as a random effect. Dot shapes represent 3 different human donors and mean lines show the mean of 3 independent stimulations or infections per donor for the control condition. (A) Cytokine protein secretion for the media only, TB WCL, and live Mtb, stimulation conditions are plotted according to dose of AACOCF3 pretreatment. (B) Cytokine protein secretion for the media only, and live Mtb stimulation conditions for the *SLC1A1* and *SLIT3* siRNA sequences compared to Negative control siRNA. (C) Fold change of Mtb-induced cytokine expression for the media only, and live Mtb stimulation conditions for the *SLC1A1* and *SLIT3* siRNA sequences compared to Negative control siRNA. (D) Cytokine protein secretion for the media only and Mtb stimulation conditions after N-SLIT3 (N), C-SLIT3 (C), and N&C-SLIT3 (N&C) pretreatment compared to Vehicle only (V) control. (E) Mtb-mCherry surface area within infected macrophages

at 4, 24, 48, and 72 hour post infection timepoints after N-SLIT3 (N), C-SLIT3 (C), and N&C-SLIT3 (N&C) pretreatment compared to Vehicle only (V) control. Results show consistent differences in cytokine production and control of intracellular Mtb replication for significant outcomes within donor infections despite baseline differences in control means between donors. Adjustment of donor as a random effect allows for significance assessment and visualization of consistent biological effect across individual infections and donors despite baseline mean differences.

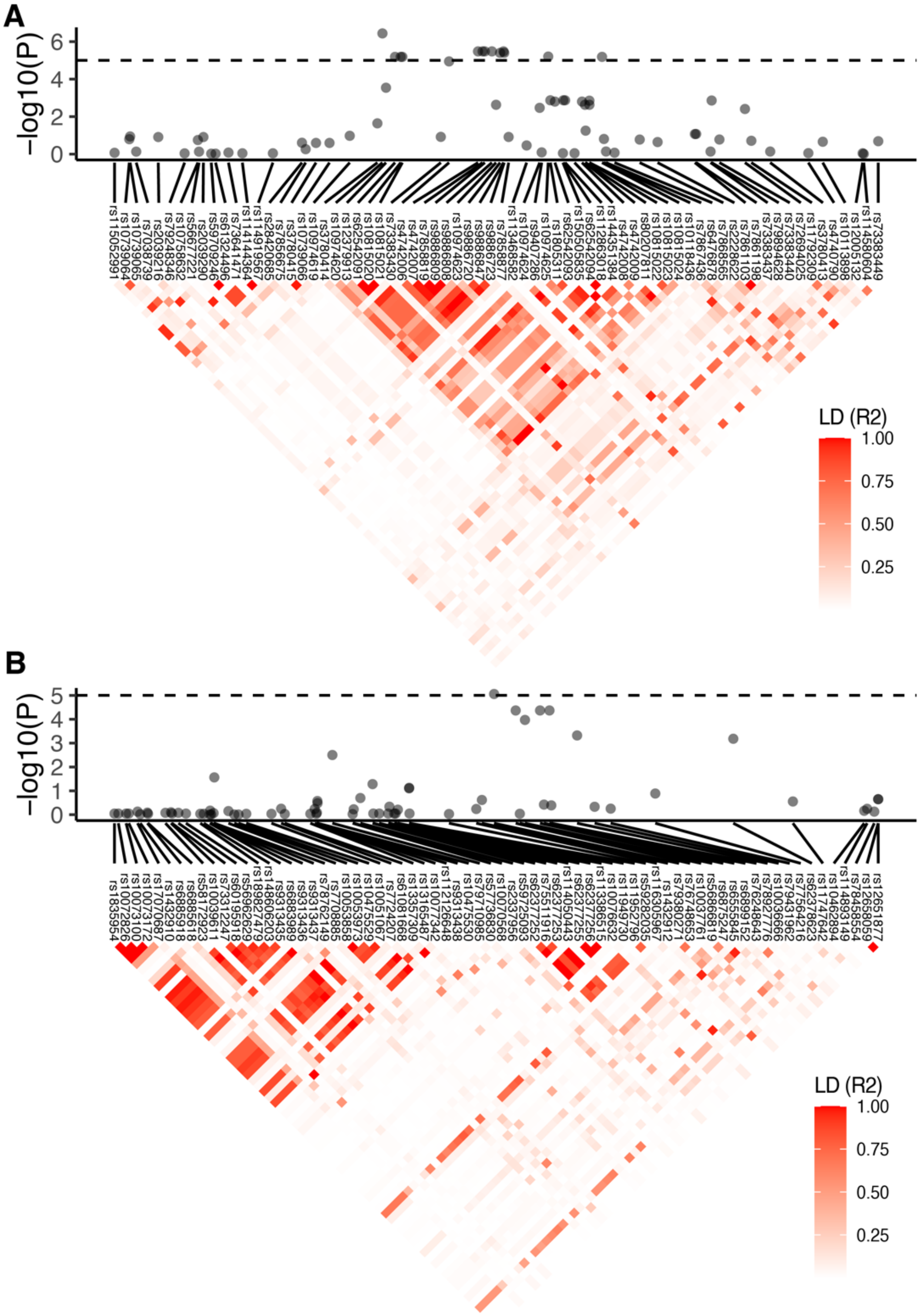
A
-log10(P)
LD (R2)
1.00
0.75
0.50
0.25
B
-log10(P)
LD (R2)
1.00
0.75
0.50
0.25

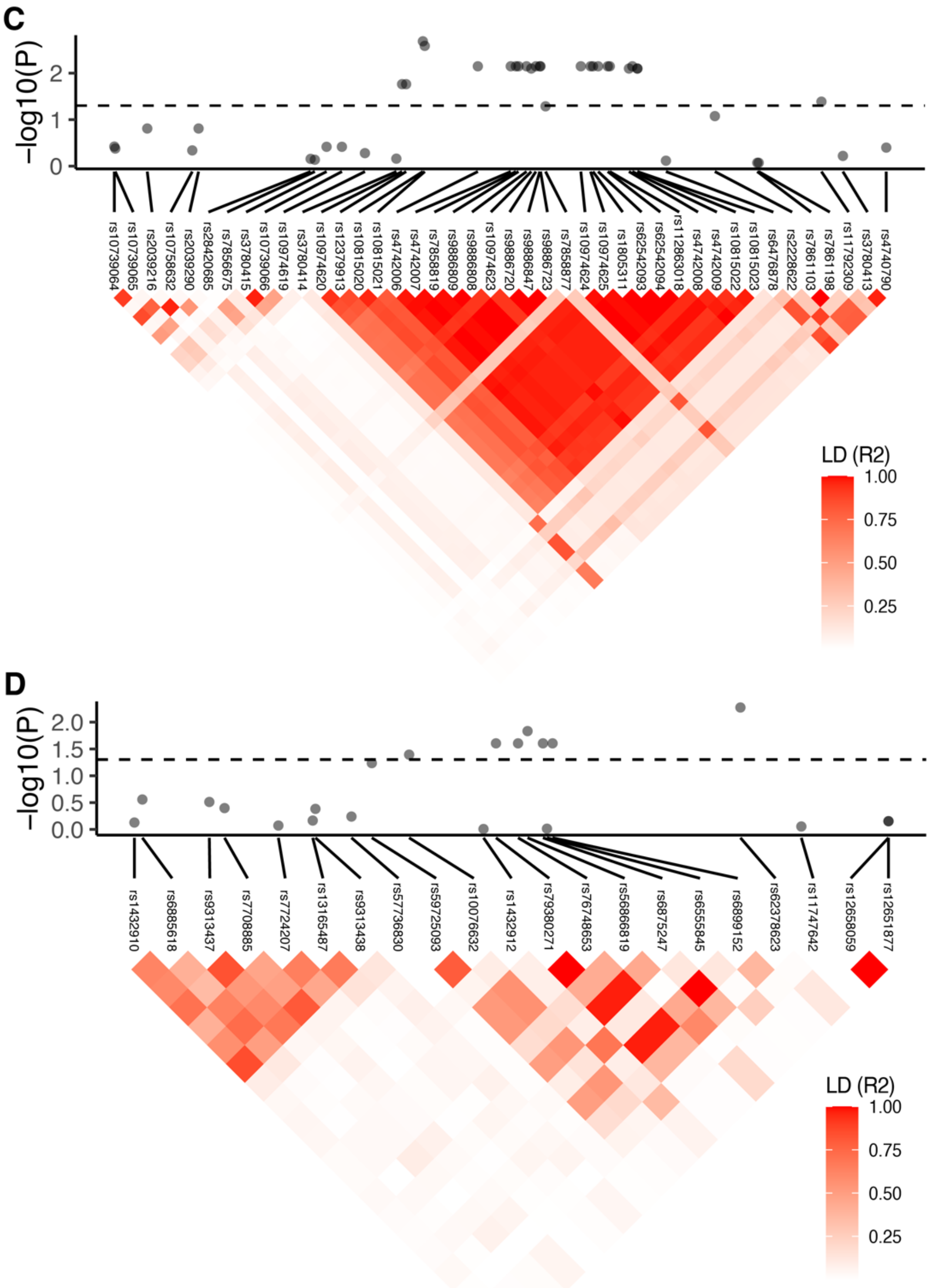

Supplemental Figure 7: LD plots of the *SLC1A1* and *SLIT3* loci in Uganda and Seattle. *P*-values of SNP associations with trait (Mtb-induced cytokine expression) are plotted with corresponding LD heatmap for the (A) *SLC1A1* in Uganda, (B) *SLIT3* in Uganda, (C) *SLC1A1* in Seattle and, (D) *SLIT3* in Seattle, loci. Despite differences in LD patterns between populations, multiple SNPs in linkage with the lead SNPs of each locus show significant associations in both populations.

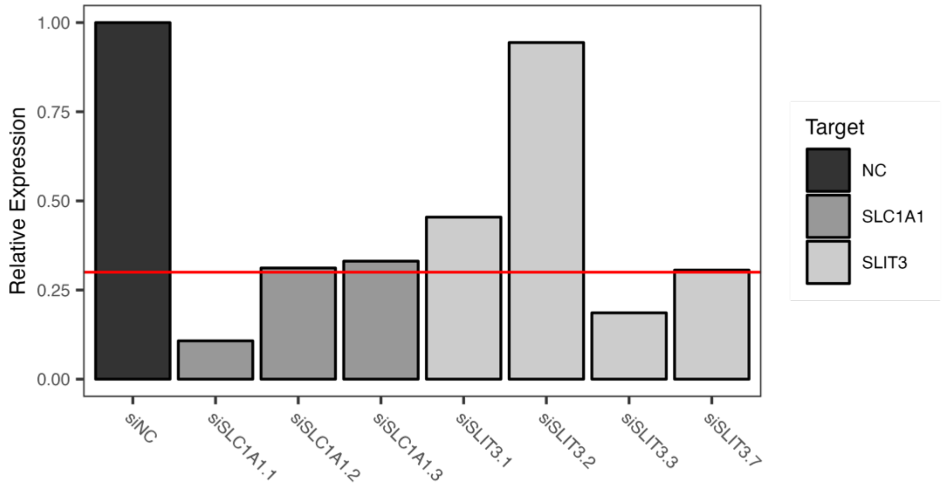

Supplemental Figure 8: Knockdown of gene expression relative to Negative Control (NC) siRNA. Three siRNA per gene originally screened were tested for sufficient gene knockdown (>70% indicated by red line) for *in* vitro studies. Shows *SLC1A1* siRNA 1 and *SLIT3* siRNA 3 should be selected for initial studies.

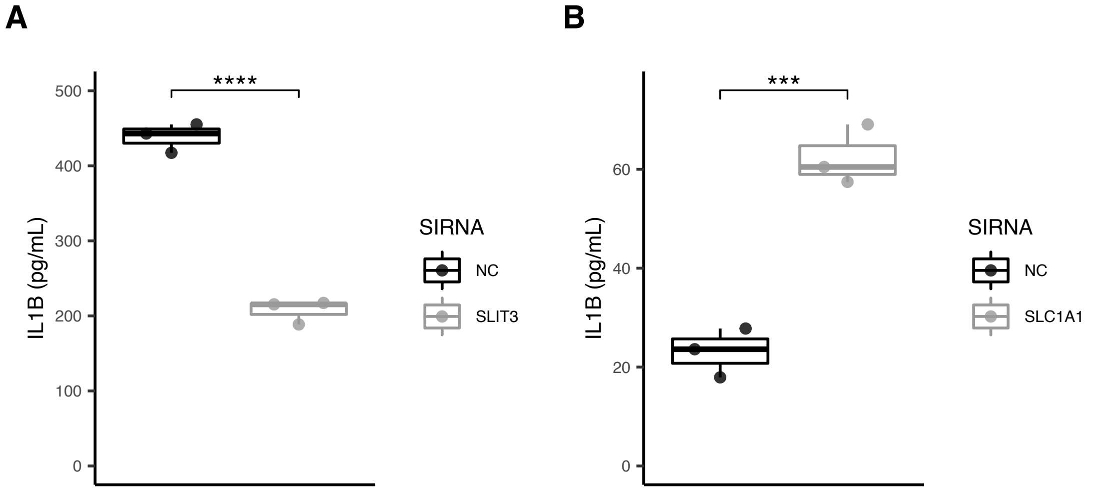

Supplemental Figure 9: Effect of additional siRNA on Mtb-induced IL1B protein secretion. (A) An independent *SLIT3* validation siRNA and (B) *SLC1A1* validation siRNA show consistent effect on Mtb-induced IL1B protein secretion when repeated with a unique siRNA sequence. Results shown are from 3 biological replicates in 1 human donor per siRNA or treatment. Significance assessed using simple linear model Significance determined as (*** $P < 0.001$, **** $P < 0.0001$).

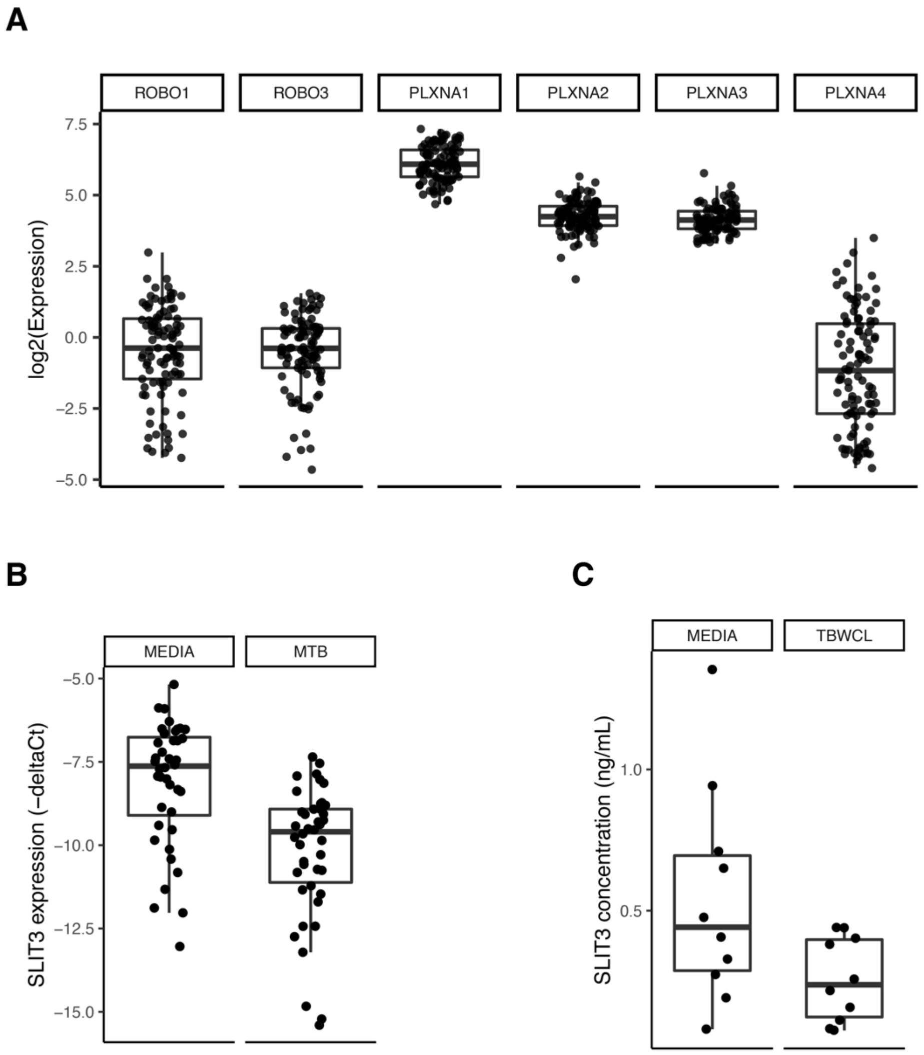

Supplemental Figure 10: Expression of queried genes. (A) Expression of queried genes in the Uganda cohort (n = 100) with detectable expression in RNAseq for the ROBO and PLEXIN A family members. Receptors with observed expression were plotted as log2(counts per million) (B) Expression of *SLIT3* from 40 human donor MDMs with Mtb and Media stimulation assessed by qPCR shows significant decrease in *SLIT3* relative to GAPDH control after 6 hours of Mtb stimulation ($P = 6.32 x 10^{-6}$). (C) ELISA assessment of SLIT3 protein abundance in 10 human donor MDMs with TBWCL and Media stimulation shows strong SLIT3 protein expression that is significantly downregulated after Mtb stimulation (*P = 0.043)*.

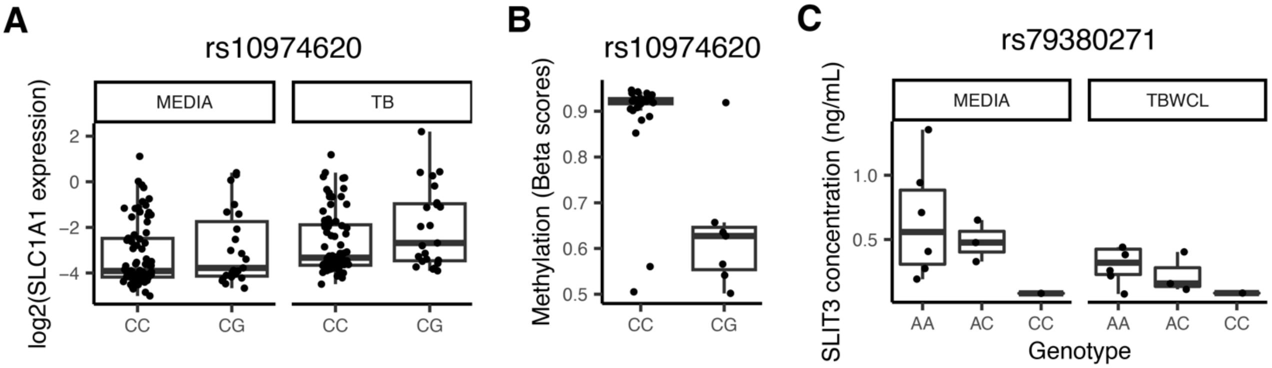

Supplemental Figure 11: *SLC1A1* and *SLIT3* lead SNP assessment of QTL associations. (A) Expression of *SLC1A1* in Ugandan cohort monocyte RNAseq (n = 100) after 6 hours of Mtb and Media stimulation show a significant increase in *SLC1A1* Mtb-expression for individuals heterozygous for the rs10974620 uncommon allele (*P = 0.0085*). (B) Methylation beta values of unstimulated Ugandan cohort monocytes (n = 40) show validate a highly significant decrease in methylation levels at CpG location chr9:4559892 previously shown in iMethyl for the rs10974620 SNP ($P = 4.2x10^{-9}$). (C) Lysate protein abundance of SLIT3 assessed by ELISA in 10 Seattle MDM donors suggest that increasing uncommon allele frequency of rs79380271 may be associated with decreased levels of SLIT3 protein after Media and TBWCL stimulation (*P* = 0.198 and 0.169 respectively). rs10974620 significance assessed using mixed linear model adjusting for genotypic pc1, pc2, sex, age, experiment and kinship. Rs79380271 assessed using simple linear model.

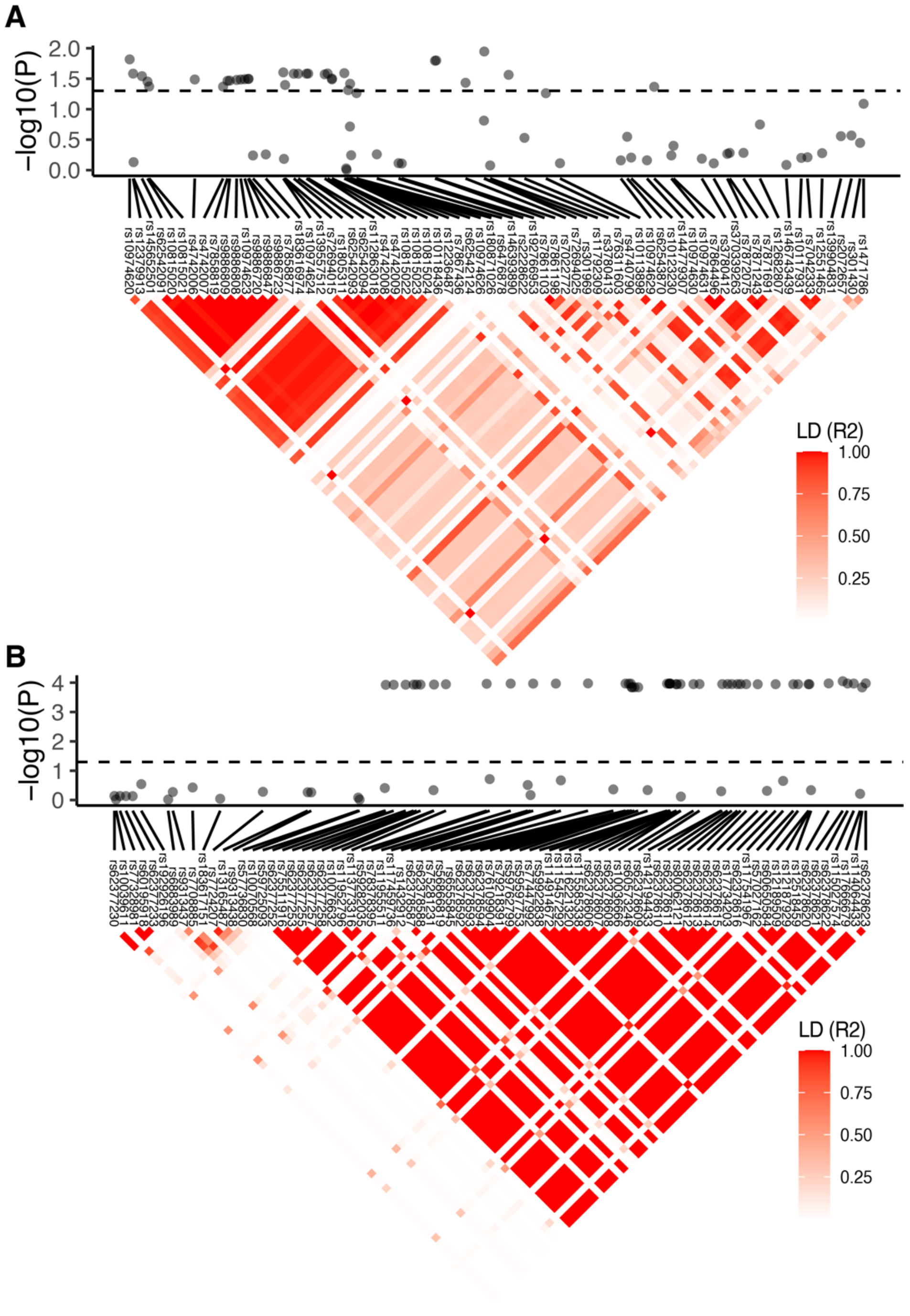

Supplemental Figure 12: LD plots of the *SLC1A1* and *SLIT3* loci in Vietnam. *P*-values of SNP associations with traits are plotted with corresponding LD heatmap for (A) *SLC1A1* TBM association and, (B) *SLIT3* TBM survival association within the Vietnam population. Significant SNPs for the *SLC1A1* and *SLIT3* loci are in high LD with many adjacent SNPs which also show similar levels of significance.

Supplemental Tables:

Supplemental Table 1: Cohort tables of Donor samples and Clinical TB phenotypes: Donor characteristics of individuals assessed within the Uganda GWAS, Seattle, Uganda TST/IGRA conversion, Vietnam Pulmonary TB, and Vietnam TBM cohorts.

Supplemental Table 2: All Functionally Annotated Suggestive Genomic Loci Associated with Mtb-Induced Monocyte Cytokines: all 77 loci with a suggestive *P*-value (1×10^{-5}) across all cytokines shown with lead SNP *P*-values, mapped genes, number of linked significant SNPs in loci at 0.01, 0.001, and 0.00001 thresholds, significance of media cytokine effect, significant cis-eQTL associations, annotated CADD scores, 1000 Genomes population allele frequencies, and GWAS catalog associations.

Supplemental Table 3: Results of SNPs tested for population spanning effect in Seattle cohort: All lead SNPs within each suggestive genomic locus were tested for association with Mtb-induced cytokine expression in the Seattle cohort using a linear mixed model in GENESIS, if SNPs with sufficient allele frequency were available. Results separated by cytokine and shown with unadjusted nominal *P*-value as well as effect size within each population.
